# Temporal fruit microbiome and immunity dynamics in postharvest apple (*Malus* x *domestica*)

**DOI:** 10.1093/hr/uhaf063

**Published:** 2025-02-25

**Authors:** Roselane Kithan-Lundquist, Hannah M McMillan, Sheng-Yang He, George W Sundin

**Affiliations:** Department of Microbiology, Genetics, and Immunology, Michigan State University, East Lansing, MI, USA; Department of Biology, Duke University, Durham, NC, USA; Department of Microbiology, Genetics, and Immunology, Michigan State University, East Lansing, MI, USA; Department of Energy Plant Research Lab, Michigan State University, East Lansing, MI, USA; Howard Hughes Medical Institute, Duke University, Durham, NC, USA; Department of Plant, Soil, and Microbial Sciences, Michigan State University, East Lansing, MI, USA; Department of Plant, Soil, and Microbial Sciences, Michigan State University, East Lansing, MI, USA

## Abstract

The plant immune response plays a central role in maintaining a well-balanced and healthy microbiome for plant health. However, insights into how the fruit immune response and the fruit microbiome influence fruit health after harvest are limited. We investigated the temporal dynamics of the fruit microbiota and host defense gene expression patterns during postharvest storage of apple fruits at room temperature. Our results demonstrate a temporal dynamic shift in both bacterial and fungal community composition during postharvest storage that coincides with a steep-decline in host defense response gene expression associated with pattern-triggered immunity. We observed the gradual appearance of putative pathogenic/spoilage microbes belonging to genera *Alternaria* (fungi) and *Gluconobacter* and *Acetobacter* (bacteria) at the expense of *Sporobolomyces* and other genera, which have been suggested to be beneficial for plant hosts. Moreover, artificial induction of pattern-triggered immunity in apple fruit with the flg22 peptide delayed the onset of fruit rot caused by the fungal pathogen *Penicillium expansum*. Our results suggest that the fruit immune response helps to orchestrate a microbiome and that the collapse of the immunity results in the proliferation of spoilage microbes and fruit rot. These findings hold implications for the development of strategies to increase fruit quality and prolong shelf life in fruits and vegetables.

## Introduction

In recent years, the microbiome is viewed as a driver of some of the physiological capabilities of eukaryotic hosts [1, 2]. In plants, members of the microbiome can live as epiphytes on the surface of the plant tissue and as endophytes within the plant tissue in the rhizosphere (belowground tissue), the phyllosphere (aboveground tissue), and the carposphere (the fruit tissue) [3, 4]. The plant microbiome is a key determinant of plant health and productivity, and its composition is driven by the surrounding environment, host compartment, host genotype, host immunity and microbe-microbe interactions [5, 6]. Highly complex, yet stable, microbial communities that are associated with numerous plants have been identified and are distinct across tissue types and geographic locations [7]. Within the plant microbiome, individual organisms confer fitness advantages that promote essential processes including growth and development, abiotic stress tolerance, nutrient uptake, and pathogen resilience [8, 9]. Niche adaptation and environmental conditions may play a major role in which microbes have beneficial functions, where only successful colonizers that can compete for the available resources or microbial groups that can adapt to the plant environment will flourish [9].

The importance of the plant immune system in the establishment and maintenance of the microbiome has been illustrated in recent studies. Pattern-triggered immunity (PTI) is the first line of inducible defense of plants against pathogens [10]. PTI is triggered via activation of pattern-recognition receptors (PRRs) upon binding of conserved microbial molecular signatures such as flagellin, lipopolysaccharides, chitin and elongation factor TU-derived peptides, referred to collectively as pathogen- (or microbe-) associated molecular patterns (PAMPs/MAMPs). The perception of MAMPs by PRRs induces complex signaling pathways that result in numerous cellular changes [11]. [12] demonstrated that an immune-compromised *Arabidopsis thaliana* quadruple mutant, affected in PTI and MIN7-asscoiated vesicle traffic, displayed bacterial dysbiosis of the leaf endophytic microbiome. Similarly, studies have shown that Arabidopsis mutants defective in the NADPH oxidase RBOHD [13] or the phytosulfokine receptor 1 (PSKR1) [14] display dysbiosis in leaf and root microbiomes, respectively. In all of these cases, dysbiosis was associated with overall poor health of the plant highlighting the significance of a balanced microbiome for plant health and the central role plant immunity can play in maintaining a balanced microbiome.

Postharvest losses of fruits and vegetables represent an enormous drain on the global food supply and could be in part the result of a breakdown in the relationship between fruit immune responses, the microbiome, and fruit health. Indeed, the Food and Agriculture Organization of the United Nations states that over one-third of food produced for human consumption is lost or wasted between production and consumption [15]. Factors contributing to loss or waste include product deterioration, physiological disorders, and excess perishable products due to microbial infection [16]. In contrast to leaf and root microbiomes, our knowledge of postharvest fruit immune responses and of the fruit microbiome is very limited. Apple represents the most consumed fruit worldwide and provides an excellent model for the study of fruit microbiomes, fruit immunity and their relationship to postharvest losses.

We hypothesized that developing and freshly harvested fruits would possess a robust innate immunity response that maintains a healthy microbiota to prevent fruit decay. However, the resources necessary to support innate immunity in fruit after harvest are likely to be gradually depleted over time. This could lead to a disruption of healthy microbiota, resulting in the proliferation of pathogenic and opportunistic microbes. A proper understanding of how the fruit microbiota is established and maintained could facilitate an untapped approach to enhance fruit health, defense, and productivity. Indeed, recent studies have demonstrated that management practices [17, 18], geographical locations [19], postharvest treatments [20], genetic background [21], and cold storage systems [22] impact microbial community composition and diversity on postharvest apple fruits. It has also been shown that microbial communities of apple fruit undergo temporal changes during cold storage over the course of months. However, the underlying mechanism(s) remain unclear.

In this study, we sought to investigate the temporal dynamics of the fruit microbiota and fruit defense gene expression during postharvest storage of apple fruits at room-temperature and to relate these dynamics to fruit decay. By tracking epiphytic and endophytic microbial community composition, as well as host PTI-associated gene expression, we found that the apple fruit microbiota is highly dynamic during postharvest storage and that expression of PTI-associated genes decreases over time. These patterns were observed in two apple cultivars, ‘Gala’ and ‘Jonathan’, despite their differing genetic makeup and microbial community composition. To our knowledge, this study represents the first exploration of the apple fruit microbiota and apple fruit PTI gene expression patterns under the natural progression of fruit spoilage.

## Results

### Fungal communities associated with pulp and skin tissues in gala and Jonathan

**Figure 1:**
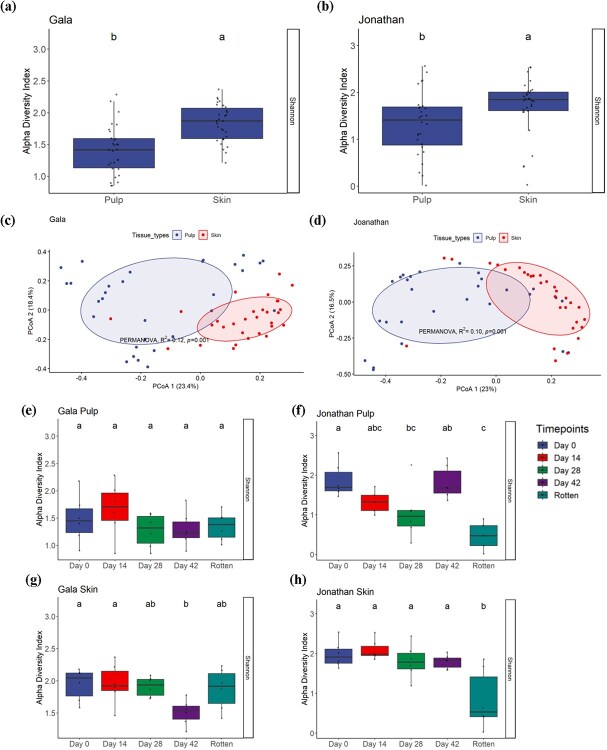
(a) and (b), Box plots showing fungal α-diversity based on the Shannon index between pulp and skin. Superimposed on the box plots are the horizontally jittered raw data points. The different letters above each bar indicate statistical significance at *P*_adj_ < 0.05 as calculated by Tukey's HSD test. (c) and (d) Principal coordinates (PCo) analysis based on Bray–Curtis dissimilarity metrics comparing fungal communities between pulp and skin. Box plots showing fungal α-diversity based on the Shannon index between time points in (e) Gala pulp, (f) Jonathan pulp, (g) Gala skin, and (h) Jonathan skin. The different letters above each bar indicate statistically significant differences at *P*_adj_ < 0.05 as calculated by Tukey's HSD test

To evaluate the effects of apple fruit tissue on fungal diversity, we performed ITS gene amplicon sequencing on the skin and pulp separately. Our results showed that apple fruit tissue type had a significant effect on fungal diversity in both ‘Gala’ and ‘Jonathan’, as indicated by the Shannon index (Fig. 1a and b). The fungal diversity present in the apple skin was significantly higher compared to the pulp tissues.

A principal coordinate analysis (PCoA) also illustrated the distinct fungal community composition in the different tissue types based on Bray–Curtis dissimilarity distance (Fig. 1c and d). The fungal community composition differed significantly between apple skin and pulp (PERMANOVA: Gala: *R*^2^ = 0.12, *P* = 0.001 and Jonathan: *R*^2^ = 0.10, *P* = 0.001). Differences in fungal community composition between tissue types were also observed in both cultivars (Fig. 1c and d).

### Temporal dynamics of fungal diversity and community structures in postharvest apple fruit

We observed significant alterations in fungal α-diversity over the course of postharvest storage in ‘Jonathan’. In ‘Jonathan’, apples at the rotten stage in both tissue types harbored the lowest fungal α-diversity according to the Shannon index (Fig. 1f and h). However, this trend was not observed in ‘Gala’, where ‘Gala’ skin showed a significant decrease in diversity at day 42, and ‘Gala’ pulp did not show any change in α-diversity over the course of time according to the Shannon index (Fig. 1e and g).

**Figure 2:**
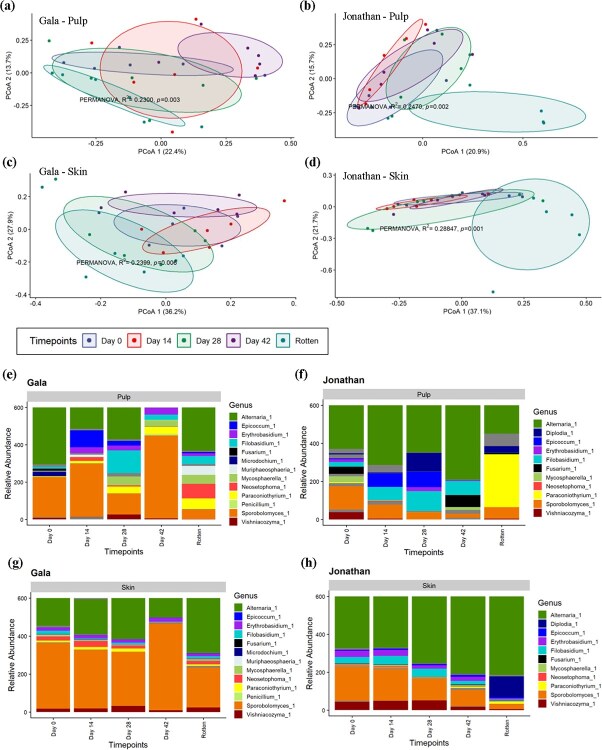
PCoA based on Bray–Curtis dissimilarity metrics comparing the fungal communities between time points in, (a) Gala pulp, (b) Jonathan pulp, (c) Gala skin, and (d) Jonathan skin. Bar plots representing mean relative abundance of the most prevalent fungal genera present across timepoints. (e) Gala Pulp, (f) Jonathan Pulp, (g) Gala Skin, and (h) Jonathan Skin. Grey color shade in the bar plot represents fungal taxa with ≤1% abundance

Additionally, the apple fruit fungal community composition during postharvest storage showed a significant change in Beta diversity over time on both cultivars and in both tissue types (PERMANOVA: Gala pulp: *R*^2^ = 0.24, *P* = 0.007, Jonathan pulp: *R*^2^ = 0.30, *P* = 0.002, Gala skin: *R*^2^ = 0.22, *P* = 0.01, and Jonathan skin: *R*^2^ = 0.31, *P* = 0.003). A principal coordinate analysis (PCoA) of the fungal community illustrates the distinct fungal community composition in the different tissue types based on Bray–Curtis dissimilarity distance (Fig. 2a-d).

The pairwise comparison between time points showed a significant change in fungal community composition starting at day 42 after harvest in both pulp (Fig. S1b) and skin (Fig. S2b) tissue in ‘Jonathan’. Similarly, in ‘Gala’ pulp a significant difference in fungal community was observed starting at day 42 (Fig. S1a). However, the change in fungal community composition in ‘Gala’ skin was observed only at rotten stage (Fig. S2a).

### Relative abundance of fungal microbial taxa over time after harvest

The relative abundance of fungal genera detected across all samples is shown in Fig. 2e-h. Overall, the abundance of *Alternaria* was seen to gradually increase over the course of postharvest storage in the skin. The shift in the relative abundance of fungal genera in pulp showed a more rapid change over the experimental time course than in the skin. This observation was very similar in ‘Gala’ and ‘Jonathan’ despite being distinct cultivars with Gala having a greatly slower rate of decay. The second most abundant fungal genus in skin was *Sporobolomyces*. The relative abundance of *Sporobolomyces* was also high at several timepoints in Gala pulp and at day 0 in Jonathan pulp. There was a striking decrease in the relative abundance of *Sporobolomyces* that coincided with the increase of *Alternaria* relative abundance over time in Jonathan skin (Fig. 2h). This was observed also in Gala skin, though the trend is not as strong (Fig. 2g). In pulp samples, changes in the relative abundance of *Alternaria* and *Sporobolomyces* were more stochastic, though the relative abundance of *Sporobolomyces* did generally decrease over time (Fig. 2e and f). *Vishniacozyma* was observed across all samples even though their relative abundance was substantially less than *Alternaria* or *Sporobolomyces*. *Filobasidium* was also observed across all samples with a higher relative abundance in the earlier time points than the later time points in the skin samples. The shift in relative abundance of fungal genera in the skin tissue was slower but steadier and more consistent than in the pulp where the change in abundance and presence and absence of different genera appeared more stochastic.

Hierarchical clustering revealed several taxa with similar trends in abundance over time. Some *Sporobolomyces* OTUs were observed at high relative abundance at earlier timepoints when the apples were healthy in both tissues and cultivars (Fig. S3). In ‘Gala’ pulp, *Sporobolomyces patagonicus* and *Filobasidium* decreased in abundance dramatically at the rotten stage while OTUs in the genera *Alternaria*, *Paraconiothyrium*, *Neosetophoma*, and *Mycosphaerella tassiana* all increased in abundance at the rotten stage (Fig. S3a). In ‘Jonathan’ pulp, *Paraconiothyrium brasiliense, Colletotrichum,* and *Fusarium* increased in abundance at the rotten stage while *Sporobolomyces, Entyloma* and *Filobasidium* all show a relative decrease (Fig. S3c). In ‘Jonathan’ skin, the genus *Alternaria*, *Colletotrichum*, and *Diplodia* gradually increased in abundance at rotten stage, while *Sporobolomyces patagonicus* and *Filobasidium* gradually decreased in abundance (Fig. S3d). No indicator species were identified for any time point in both ‘Gala’ and 'Jonathan’. Differentially abundant taxa that were identified using DESeq2 and indicator species analysis at the sampling time points in Gala and Jonathan is shown in Table S1.

### Bacterial communities associated with pulp and skin tissues in gala

**Figure 3:**
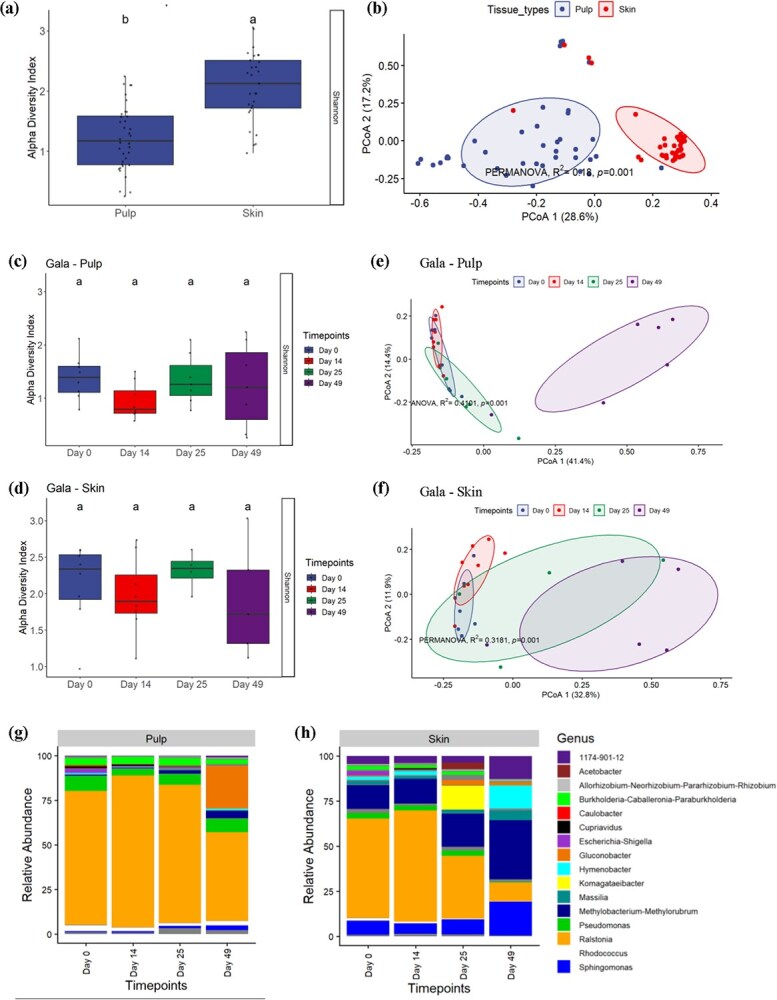
Bacterial communities associated with pulp and skin tissues in Gala. (a) Box plots showing the bacterial α-diversity based on the Shannon index between pulp and skin tissues, (b) PCoA based on Bray–Curtis dissimilarity metrics, showing the distance in the bacterial community composition between pulp and skin. Effect of postharvest aging on bacterial diversity, and community structures in Gala fruit. (c) and (d) box plots showing the bacterial α-diversity based on the Shannon index between time points in Gala pulp and skin, respectively. The different letters above each bar indicate statistically significant difference at *P*_adj_ < 0.05 as calculated by Tukey's HSD test. (e) and (f) PCoA based on Bray–Curtis dissimilarity metrics, showing the distance in the bacterial communities between timepoints in Gala pulp and skin, respectively. (g) and (h) Relative abundance of the most prevalent bacterial genera present across sampling points at postharvest in Gala Pulp and Skin, respectively. Grey color shade in the bar plot represents bacterial taxa with ≤1% abundance

Apple skin and pulp harbored significantly different levels of bacterial diversity. Like in fungi, apple skin also harbored a significantly higher bacterial alpha diversity than the pulp according to the Shannon index (Fig. 3a). Additionally, the bacterial community composition differed significantly between apple skin and pulp as analyzed by Bray–Curtis dissimilarity distances (PERMANOVA: Gala: *R*^2^ = 0.18, *P* = 0.001). The distinct bacterial community composition between the tissue types in ‘Gala’ apple is shown in Fig. 3b.

### Temporal dynamics of bacterial diversity, community structures, and relative abundance in postharvest apple fruit

The apple fruits at postharvest did not show any change in bacterial alpha diversity over the sampling time points (day 0 to day 49) (Fig. 3c and d). The bacterial diversity result for ‘Gala’ skin contrasted with the fungal diversity in that it showed a significant decrease in diversity at day 42 (Fig. 1g), although with a caveat that the samples were collected in different years. However, similar to the fungal community composition, there was a dynamic shift in bacterial community composition in both tissues (PERMANOVA: ‘Gala’ pulp: *R*^2^ = 0.4101, *P* = 0.001, ‘Gala’ skin: *R*^2^ = 0.3181, *P* = 0.001). The distinct bacterial community composition over postharvest storage was illustrated by PCoA based on Bray–Curtis dissimilarity distance (Fig. 3e and f).

There was a shift in relative abundance of bacterial genera over time in both tissues, much like was seen with the fungal community composition. Although the relative abundances of bacterial genera in the pulp do not change significantly until Day 49, the bacterial community composition changes substantially over time in the skin (Fig. 3g and h). These findings support the result from the Bray–Curtis dissimilarity distances in skin and pulp, where the community in pulp is most dissimilar at Day 49 (Fig. 3e) and the skin shows a gradual change across time points (Fig. 3f). The most abundant genus observed across both tissues was *Ralstonia*. The abundance of *Ralstonia* in apple fruit microbiome was also observed in a study conducted by Wassermann et al. 2019a and 2019b. There was also a high abundance of *Methylobacterium* in the skin tissue that showed a gradual increase over time. *Methylobacterium* was also present in the pulp and showed a similar increase in abundance over time in the pulp, albeit with a lower relative abundance. *Sphingomonas* and 1174–901-12 were also observed in moderate abundance in skin with an increase in relative abundance over time. Some other genera that were present across all samples are *Burkholderia*, *Pseudomonas,* and *Rhodococcus*. However, some of these showed a decrease in abundance over time, especially in skin. *Gluconobacter* was seen in high relative abundance at day 49 in the pulp sample and also showed an increase in abundance at day 25 and day 49 in the skin samples. Overall, there was a shift in relative abundance of the most prevalent as well as the less prevalent genera overtime.

Hierarchical clustering revealed that *Caulobacter, Hymenobacter, Cupriavidus*, and *Pseudomonas* genera were abundant in healthy apples (day 0 and day 14) but steadily declined in relative abundance over the course of postharvest in both tissues (Fig. S4). Interestingly, OTUs belonging to *Gluconobacter* and *Acetobacter* genera, some members of which were reported to cause apple fruit decay [23], began appearing at 49 days postharvest (Fig. S4). In ‘Gala’ pulp, a total of 24 indicator species were identified at days 25 and 33 indicator species at day 49. Table S2 shows the differentially abundant taxa that were identified at each time point in pulp and skin, using DESeq2 and indicator species analysis.

### Network analysis reveals possible drivers of compositional changes during postharvest

To identify possible keystone taxa, connectors, and hubs that may drive changes in apple microbiome composition during postharvest storage, we performed network analysis using OTU abundance over time for each sample group (Figs S5a, S5b, S6a, S6b, S7a, S7b, S8a, S8b, S9a, S9b, S10a, S10b and Tables S1-S6). Overall, all generated networks were sparse, with each OTU connecting on average to only two other OTUs (Table S5). For fungal communities, pulp networks were slightly denser than skin communities and showed higher overall network centralization (Table S5). In contrast, bacterial communities in ‘Gala’ pulp and ‘Gala’ skin were extremely similar, with identical measures of network density and very similar measures of centralization (Table S5). Most associations identified in all networks were positive; however, fungal communities in ‘Gala’ skin and ‘Jonathan’ pulp had a notably higher ratio of negative to positive associations (Figs S6a, S6b, S7a, S7b and Table S5).

**Figure 4:**
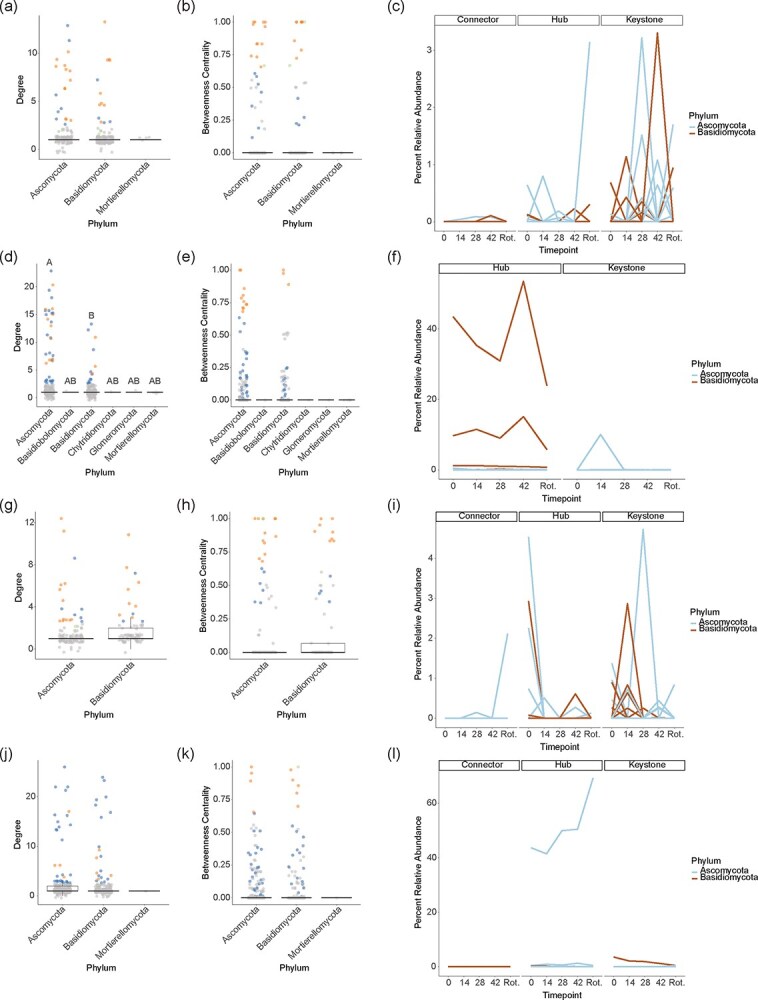
Network analysis for fungal communities in apple fruit. Scatter points show the degree (a, d, g, j) or betweenness centrality (b, e, h, k) calculated for each network node. Hub, keystone, and connector nodes are indicated. Network measurements were compared across phyla with ANOVA. No significant differences were detected except in ‘Gala’ Skin (d). For ‘Gala’ Skin (d), differences between phyla were compared using a Tukey HSD post hoc test. Different letters indicate statistical significance (*P*_adj_ < 0.05). Enrichment of hubs, keystones, and connectors were calculated and compared for each phylum using a Fisher Exact Test with Benjamini Hochberg correction. No phyla were enriched for any node type. (c, f, i, l) Traces show percent relative abundance across timepoints for individual nodes. Roles as connectors, hubs, or keystones are indicated at the top of each plot. Different phyla are indicated. (a-c) ‘Gala’ Pulp. (d-f) ‘Gala’ Skin. (g-i) ‘Jonathan’ Pulp. (j-l) ‘Jonathan’ Skin

**Figure 5:**
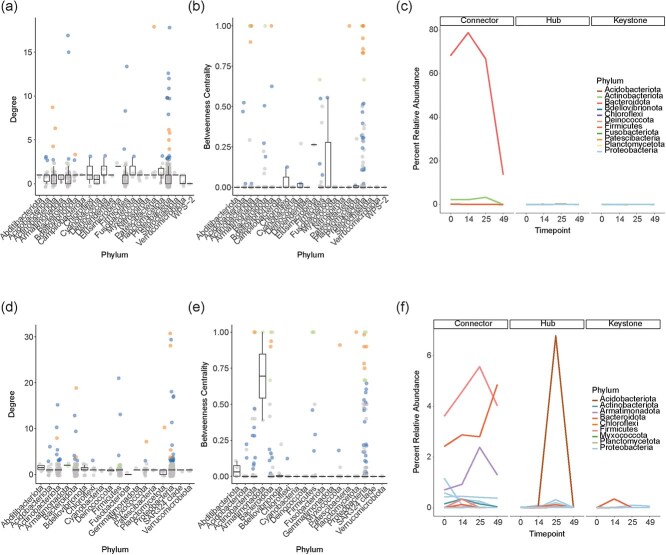
Network analysis for bacterial communities in apple fruit. Scatter points show the degree (a, d) or betweenness centrality (b, e) calculated for each network node. Hub, keystone, and connector nodes are indicated. Network measurements were compared across phyla with ANOVA. No significant differences were detected. Enrichment of hubs, keystones, and connectors were calculated and compared for each phylum using a Fisher Exact Test with Benjamini Hochberg correction. No phyla were enriched for any node type. (c, f) Traces show percent relative abundance across timepoints for individual nodes. Roles as connectors, hubs, or keystones are indicated at the top of each plot. Different phyla are indicated. (a-c) ‘Gala’ Pulp. (d-f) ‘Gala’ Skin

As a result of the sparse network structures, many OTUs were identified as hubs with fewer characterized as connectors in each cultivar and tissue type for fungal and bacterial communities (Figs S5c, S6c, S7c, S8c, S9c, S10c and Tables S1 and S2). Interestingly, there were still a substantial number of OTUs characterized as keystone taxa with both a high betweenness centrality and high degree (Figs S5c, S6c, S7c, S8c, S9c, S10c and Tables S1 and S2). This could reflect small, yet highly interconnected network clusters with loop structures. To probe these features further, we clustered the network and determined the percentage of nodes in each cluster belonging to distinct phyla (Figs S5d, S6d, S7d, S8d, S9d, S10d and Tables S1 and S2). Most network clusters were small, with only 5–19 clusters per cultivar and tissue type containing 10 or more nodes (Figs S5d, S6d, S7d, S8d, S9d, S10d and Tables S1 and S2). Generally, the percentage of nodes in each phyla reflected the overall community structure, which suggests that clusters are relatively diverse (Figs S5d, S6d, S7d, S8d, S9d, S10d and Tables S1 and S2). We also noted that keystone, hub, and connector nodes were not limited to any one phylum, suggesting that OTUs from a variety of taxa may play important roles in determining overall network structure (Figs 4a, 4b, 4d, 4e, 4g, 4h, 4j, 4k, 5a, 5b, 5d, 5e and Tables S1-S6). One exception appeared to be the bacterial *Armatimonadota* phylum in ‘Gala’ skin, which showed a significantly higher betweenness centrality compared to other phyla but, notably, contained only two nodes (Fig. 5e and Tables S1-S6). No phyla were enriched for keystone, hub, or connector nodes in either cultivar, tissue type, or microbial community (Figs 4a, 4b, 4d, 4e, 4g, 4h, 4j, 4k, 5a, 5b, 5d, 5e and Tables S1-S6). Indeed, no phyla had a significantly higher degree or betweenness centrality in any network with the exception of fungi in ‘Gala’ skin, among which Ascomycota had a significantly higher degree than Basidiomycota (Fig. 4d and Tables S1-S6).

Perhaps most intriguing were the abundance patterns over postharvest storage timepoints among nodes identified as connectors, hubs, and keystones (Figs 4c, 4f, 4i, 4l, 5c, 5f and Tables S1 and S2). Fungal keystone taxa abundances in pulp changed dynamically over time for both ‘Gala’ and ‘Jonathan’, but the timing of increases in abundance differed between cultivars (Fig. 4c, 4f, 4i, 4l and Tables S1 and S2). For example, in ‘Gala’ pulp, fungal keystone taxa abundances increased sharply at days 28–42 before declining in the rotten stage, while in ‘Jonathan’ pulp, fungal keystone taxa increased in abundance at days 14–28, roughly two weeks before the increases in ‘Gala’ (Fig. 4c and 4i and Tables S1 and S2). Fungal hub taxa showed a rapid decline in ‘Jonathan’ pulp early during postharvest storage, while fungal hub taxa in ‘Gala’ pulp were generally more stable (Fig. 4c and 4i and Tables S1 and S2). One fungal hub taxon in ‘Gala’ pulp showed a rapid increase in abundance at the rotten stage (Fig. 4c and Tables S1 and S2). Hub taxa in skin tissue showed similarly opposite trends in ‘Jonathan’ and ‘Gala’, with ‘Gala’ skin hub taxa generally declining over postharvest storage and ‘Jonathan’ skin hub taxa increasing in abundance over storage (Fig. 4f and 4l and Tables S1 and S2). While fungal connector nodes did not show dramatic changes in abundance for ‘Gala’ or ‘Jonathan’ in either tissue type, bacterial connector nodes changed dramatically in ‘Gala’ pulp and skin, with pulp connectors decreasing in abundance at the rotten stage and skin connectors showing slight increases in abundance over postharvest storage (Fig. 5c and f and Tables S1 and S2). Peripheral nodes showed more stochastic abundance patterns over time in all networks (Figs S5e, S6e, S7e, S8e, S9e, S10e).

### Temporal expression of PTI-associated defense genes in apple fruit

**Figure 6:**
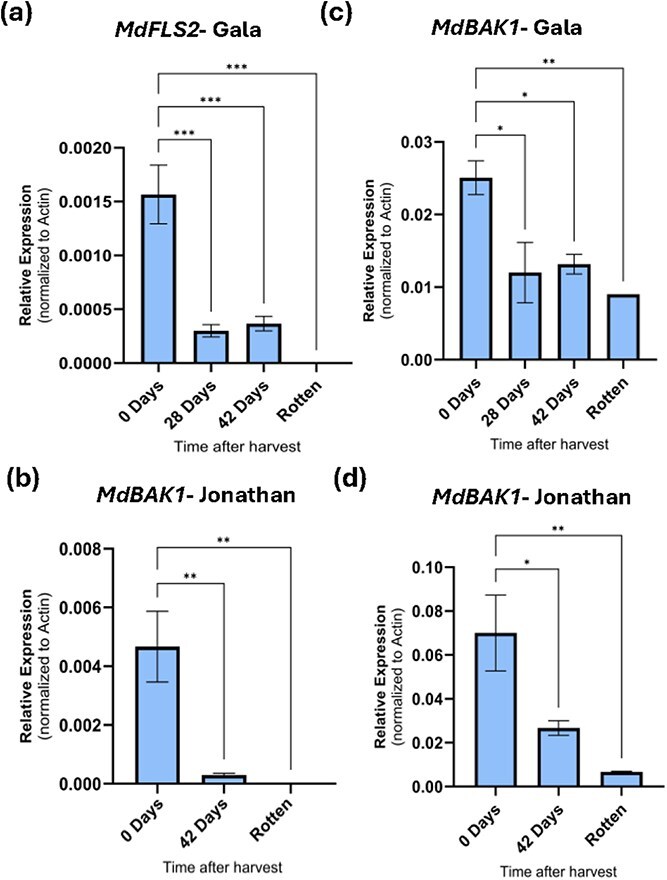
PTI gene expression in postharvest apple fruit over time. (a) *MdFLS2* Gala, (b) *MdFLS2* Jonathan, (c) *MdBAK1* Gala, and (d) *MdBAK1* Jonathan. RT-qPCR was performed with RNA isolated from apple fruits at the timepoints indicated. For RT-qPCR, transcript levels were normalized to that of the house-keeping gene (*Mdβ*-*ACTIN*). Data are means of three biological replicates. Error bars represent ±1 SE. Number of stars represent statistically significant differences between time points (One-way ANOVA, Tukey's test; ^*^P < 0.05; ^**^P < 0.01, ^***^P < 0.001)

The gene expression pattern of PRR/coreceptor genes *MdFLS2* and *MdBAK1* were investigated over the course of postharvest storage of Gala and Jonathan cultivars. Our RNA sampling was focused on timepoints close to and during dramatic shifts in microbial compositions. Accordingly, samples for *MdFLS2* and *MdBAK1* expression levels were collected only in four time points in Gala at Day 0, 28, 42, and Rotten and three time points for Jonathan at Days 0, 42, and Rotten**.** Expression of these genes was normalized to the housekeeping gene *Mdβ-ACTIN*. In both cultivars, expression of these genes decreased over time (Fig. 6). On the day of harvest, when the apple fruit was fresh and healthy, *MdFLS2* and *MdBAK1* were both highly expressed. But by day 42 after harvest, when apples had begun to dehydrate and show some signs of decay, the expression level of these genes had become significantly reduced. By the rotten stage (day 96 and day 67 for Gala and Jonathan, respectively), *MdFLS2* expression was no longer detected and *MdBAK1*, although detected, was substantially reduced compared to that at day 0 and day 42 postharvest (Fig. 6). In ‘Gala’, an additional sampling point was added to the experiment at day 28. Interestingly, there was no significant change in gene expression between day 28 and day 42, although at both time-points, the expression levels of *MdFLS2* and *MdBAK1* were significantly reduced compared to those at day 0. Overall, changes in *MdFLS2* and *MdBAK1* gene expression in apple fruit were inversely correlated to the shift in the fruit microbiota composition toward dysbiosis.

### Enhanced defense response with Flg22 treatment on postharvest apple fruits

**Figure 7:**
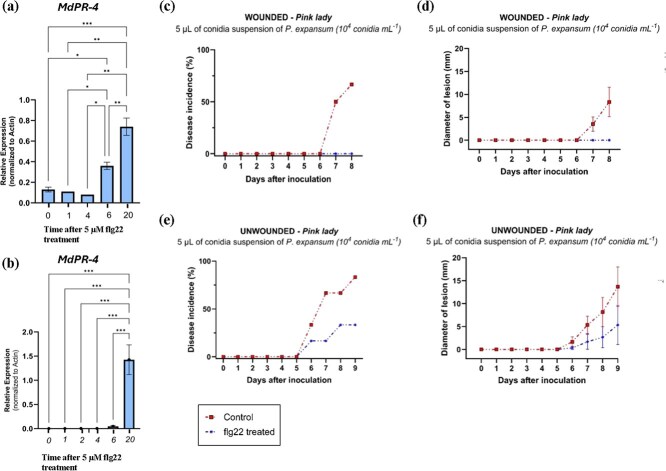
(a) and (b) Defense gene (*MdPR-4)* expression in ‘Pink Lady’ apple fruit post-flg22 treatment in skin, and Pulp, respectively. The fruits were prestabbed with a sterile needle at a width of 1 mm and a depth of 2 mm, prior to flg22 treatment. RT-qPCR was performed with RNA isolated from apple fruits at the timepoints indicated (in hours). For RT-qPCR, transcript levels were normalized to that of the house-keeping gene (*Mdβ*-*ACTIN*). Data are means of three biological replicates. Error bars represent ±1 SE. Number of stars represent statistically significant differences between time points (one-way ANOVA Tukey's test; ^*^P < 0.05; ^**^P < 0.01, ^***^P < 0.001). (c) and (d) Infection of wounded apple fruits by *P. digitatum* at 24 hrs post-flg22 treatment. Illustrating disease incidence and lesion diameter, respectively. (e) and (f) Infection of unwounded apple fruits by *P. digitatum* at 24 hrs post-flg22 treatment illustrating disease incidence and lesion diameter, respectively. Each value is the mean of six biological replicates. Error bars represent ±1 SE

To determine whether apple fruit immunity can be enhanced during postharvest, apple fruits were treated with the flg22 peptide right after harvest. flg22 is a 22-amino-acid epitope derived from bacterial flagellin and a well-characterized elicitor of PTI [24]. *MdPathogenesis-related (PR)-4* gene expression was investigated at several time points after flg22 treatment in the skin and the pulp separately. The *PR-4* gene was investigated because this gene has also been shown to have antifungal activities against fungal pathogens such as *Botryosphaeria dothidea* in apple [25] and resistance against apple replant disease pathogen [26]. RT-qPCR indicated that in the skin *MdPR-4* gene expression was significantly increased at 6 h and further enhanced by 20 h. Post-flg22 treatment (Fig. 7a). Similarly, in the pulp samples, *MdPR-4* was slightly enhanced at 6 h and very significantly enhanced by 20 h. 121668112Post-flg22 treatment (Fig. 7b). Thus, flg22 treatment had a positive impact on *PR-4* defense gene expression of the apple fruit in both tissue types within hours.

To determine the effect of flg22 treatment on apple fruit decay caused by a fungal pathogen, 5 μl of a conidia suspension of *P. expansum*, a known apple rotting fungus, was wound-inoculated in the flg22-treated and control apples 24 h post-flg22-treatment. In this experiment, the flg22 peptide was delivered through surface wounds to reach the pulp (see section Methods). We found that, in the control samples, lesions at the inoculated site became visible by 7 days postfungal inoculation. However, the flg22-treated fruits did not show any sign of infection even at eight days postfungal inoculation (Fig. 7c and d). This indicates that the induction of immunity by the flg22 peptide has a positive impact on apple fruit resistance to *P. expansum*. An additional experiment was conducted by dipping fruits in flg22-containing solution without wounding the apples. flg22 could still reduce disease incidence in the treated apples although not as effectively as delivering flg22 peptide through wounds to reach the pulp (Fig. 7e and f).

## Discussion

Fruit undergo physiological changes and eventually decay during postharvest storage, mostly due to microbial infection. The rate of postharvest decay of apple fruit varies between different cultivars [27, 28]. In this work, we investigated if there were observable patterns in the apple fruit microbiota that correlated with fruit PTI-associated gene expression after harvest. We first asked if the microbiota of the apple fruit changes during postharvest storage. We studied the change in both the endophytic (from pulp) and epiphytic (from skin) microbial communities. Consistent with Wasserman et al. [18], we observed that apple skin and apple pulp harbor distinct microbial communities. Our results also demonstrated that the apple fruit microbiota is dynamic. We observed a shift in beta diversity (Bray Curtis dissimilarity) after harvest when the fruits were stored at room temperature. Among fungi, a greater relative abundance of the genus *Sporobolomyces*, was observed in healthy fruits. Some members of *Sporobolomyces* exhibit antimicrobial activities inhibiting growth of pathogens in fruits [29]. Over the course of postharvest storage, we observed decreasing levels of *Sporobolomyces* that coincided with increasing relative abundance of the genus *Alternaria*, members of which are commonly found in a variety of habitats as ubiquitous agents of decay [30]. Interestingly, *Alternaria* became the dominant taxa in the skin of rotting apples (Fig. 3e-h). Several studies have shown that the antagonistic activity of *S. roseus* to other fungal pathogens such as *Penicillium, Alternaria* and *Aspergillus* results from degrading the mycotoxin produced by these pathogens [29, 31, 32]. *S. ruberrimus* was shown to regulate ethylene homeostasis to maintain a well-balanced availability of metals such as Fe and Ni in plants [33]. Hence, the high relative abundance of the genus *Sporobolomyces* observed in freshly harvested apple fruit could suggest a healthy microbiota that is effectively suppressing the growth of pathogens. This pattern was observed for some *Sporobolomyces* OTUs in both ‘Gala’ and ‘Jonathan’. Thus, an important future research direction is to isolate these strains and to mechanistically understand factors that contribute to the decline of *Sporobolomyces* over time.

A similar pattern was observed in the bacterial community on harvested apples where bacteria such as *Caulobacter, Cupriavidus*, and *Pseudomonas*, which contain strains that have beneficial effects on plants [34–37], were abundant in healthy apples but steadily declined in relative abundance over time (Fig. S4**).**  *Pseudomonas* species, for example, can be pathogenic, but most plant-associated *Pseudomonas* species are beneficial to plant growth and health [38]. *Pseudomonas* has also been shown to increase fruit production and protection against pests and pathogens in blackberries [39]. Members of the *Cupriavidus* and *Caulobacter* genera have been studied for their beneficial role in plant growth-promotion including production of phytohormones and metabolite synthesis [34–37]. It was also reported that genus *Hymenobacter* was found abundantly in healthy Kiwifruit plants, but its relative abundance was significantly reduced in a diseased plant [40]. We observed a similar trend in this study where the abundance of the genus *Hymenobacter* was significantly reduced in rotting apple. Our observation of these putative beneficial bacteria in healthy fruit suggests that they may be playing a role in activities contributing to beneficial fruit physiology.

Interestingly, as in fungi, *Gluconobacter* and *Acetobacter* genera began to appear in high abundance at 49 days postharvest. Members of these genera are commonly found in rotten fruits and spoiled juices and have the ability to grow at relatively low pH and low nutrient levels. These genera can also oxidize sugars and alcohols into acids [23, 41, 42]. It remains to be determined if the taxa observed in this study are involved in initiating the rotting process by changing the fruit physiology, leading to the growth of opportunistic fungal pathogens that are the ultimate causal agents of fruit rot. Overall, the fact that we observed beneficial microbes in healthy fruit, and conversely pathogens in unhealthy fruit, is consistent with the notion that the fruit microbiota plays a significant role in dictating fruit health. Of note, some of the genera, such as *Cupriavidus and Caulobacter* from healthy apples, and *Gluconobacter* and *Acetobacter* from rotting apples are rare taxa and are present in low abundance compared to other taxa in the apple microbial community. In ‘Gala’ skin, our network analysis identified 1 connector OTU and 1 hub OTU from *Cupriavidus*, 1hub OTU from *Gluconobacter*, and 1 hub OTU from *Acetobacter*. In ‘Gala’ pulp, 1 hub OTU from *Gluconobacter* was identified (Table S2). Given their key roles in the network structure, future research is needed to determine if some of these OTUs detected in our study have cascading effects in changing apple fruit microbial community dynamics [43, 44].

The abundance patterns of hub, connector, and keystone taxa over time could help explain the overall changes in alpha and beta diversity across postharvest storage timepoints and ultimately shelf-life differences between ‘Gala’ and ‘Jonathan’ cultivars. For example, ‘Jonathan’ alpha diversity in pulp and skin for fungal communities is lowest at the rotten stage (Fig. 1e-h). In pulp tissue, ‘Jonathan’ hub taxa decrease rapidly early during postharvest storage, which could destabilize the fungal community thereby allowing potentially harmful microbes to proliferate and preventing other taxa from persisting in the community (Fig. 4i). Interestingly, the three most highly abundant hubs in ‘Jonathan’ pulp at day 0 belong to *Fusarium*, *Vishniacozyma*, and *Botrytis* genera (Fig. 4i and Tables S1 and S2). Some *Vishniacozyma* species have been used as biocontrol agents in kiwifruit against *Botrytis* [45]; therefore, a decrease in this OTU may contribute to pathogen proliferation. *Vishniacozyma* also co-occurs on apple surfaces with *Penicillium expansum*, the causal agent for blue mold disease on apples [46], and in roots with *Valsa* mali, the causal agent of Valsa canker [47], though its role as beneficial or harmful microbe in these settings is not characterized. In contrast, *Fusarium* and *Botrytis* genera contain well-known plant pathogens. A decrease in nonpathogenic OTUs in these genera may leave an opening in the community for pathogens in these genera to proliferate. The most highly abundant hubs and keystones in ‘Gala’ pulp do not belong to genera with known pathogens (Fig. 4c and Tables S1 and S2).

In skin tissue, one ‘Jonathan’ hub taxon increases dramatically in abundance over time, making up over 60% of the community (Fig. 4l and Tables S1 and S2). This alone could contribute to the decline in alpha diversity and could destabilize the community structure. Additionally, this OTU is a member of the *Alternaria* genus, which contains well-known pathogenic strains [30]. In contrast, ‘Gala’ skin shows a significant decrease in alpha diversity at day 42 (Fig. 1e-h). In ‘Gala’ skin hub taxa, we observed a gradual decrease in two highly abundant hub taxa belonging to *Sporobolomyces* followed by a rapid increase at day 42 and a rapid decrease at the rotten stage (Fig. 4f and Tables S1 and S2). This boom and bust pattern could contribute to an overall collapse in community structure.

For fungal pulp keystone taxa in both ‘Gala’ and ‘Jonathan’ cultivars, abundance changes dramatically with sudden increases and decreases, but interestingly the timing of sudden increases is earlier for ‘Jonathan’ (Fig. 4c and i and Tables S1 and S2). One hypothesis is that the early peak in ‘Jonathan’ of certain keystone taxa could destabilize the community, leading to the gradual reduction in alpha diversity and systematic shift in beta diversity over time (Figs 1e-1h and 2a-2d). Perhaps this early destabilization contributes to the shorter shelf life of ‘Jonathan’. In contrast, the delayed changes in ‘Gala’ pulp keystones could suggest that the community is more stable, which could contribute to the longer shelf life observed for this cultivar.

For bacterial communities, connector taxa seem to play a larger role in community structure (Fig. 5c and f and Tables S1 and S2). Hub and keystone taxa in both pulp and skin bacterial communities have few changes in abundance over time (Fig. 5c and f and Tables S1 and S2). However, one connector in pulp rapidly decreases in later timepoints and several connector taxa increase over time in skin tissue (Fig. 5c and f and Tables S1 and S2). While this could be a result of improved ability to detect many taxa for bacteria compared to fungi, this pattern could also indicate that connectors play a larger role in bacterial communities. As these taxa change in abundance, dynamics between the various network clusters change, which may contribute to the gradual shift in beta diversity over time.

Another important finding of our study is the immune gene expression dynamics in postharvest apple fruits. Specifically, we determined that expression of the PTI receptor/co-receptor genes, *MdFLS2* and *MdBAK1*, diminished over time during postharvest storage. In healthy apples, these genes were highly expressed, but expression was significantly reduced by 42 days after harvest. In rotten apples, *MdFLS2* gene expression could not be detected, and *MdBAK1* expression was low as well. In plant innate immunity, PTI is the first line of active defense against most pathogens [10]. Recently, it has been shown that PTI is an important component that acts as a host barrier to control the level of commensal microbes and opportunistic microbes from excessive proliferation in the host tissue for optimal plant health [12–14]. In light of these observations, and our findings reported here, we hypothesize that fruit immunity likely plays an important role in maintaining a balanced and healthy microbiota and, in turn, preventing fruit spoilage. It was interesting that we observed a steep decline in the fruit immune response (as monitored by *MdFLS2* and *MdBAK1* expression) around 42 days postharvest, which coincides with the timing of the emergence of potential pathogenic microbes. We show that by inducing PTI with the flg22 peptide, we can delay the onset of fungal rot in apple fruit; thus, there seems to be an inverse relationship between PTI gene expression and microbiota dysbiosis. Future research is needed to determine if this protection is associated with composition changes of the microbiota.

Examining microbiota dynamics in fresh apple fruits during the natural ripening and decay processes presents a unique challenge for mechanistic studies. For example, because the fruits can be harvested only once per year, it is not possible to collect the microbiota from a healthy apple and a decaying apple simultaneously, apply the community to a healthy apple, and observe differences in disease protection on healthy apple fruits because healthy and decaying fruits are not available at the same time. Culturing all members of the microbial community at all-time points during postharvest storage could allow communities to be stored until the next harvest season and would enable the design of synthetic communities for further mechanistic testing. While this goes beyond the goal of our initial, foundational study, it does present an important future direction and long-term goal for postharvest storage research. Despite this limitation, our network analysis does serve to partially bridge the gap between our characterization of apple fruit microbial communities and our observed differences in apple fruit immune activity. These results could inform selection of individual or small groups of microbes for future culturing and mechanistic efforts.

To our knowledge, this study represents the first exploration of apple fruit microbiota conducted during the postharvest period and the patterns in apple fruit PTI gene expression patterns under the natural progression of fruit spoilage. Our results have implications in developing strategies to enhance fruit defense for increased fruit quality and prolonged shelf life and may motivate future studies to examine if the relationship we observed in apple fruit immunity and microbiome dynamics is generally applicable to other postharvest fruits and vegetables.

## Materials and methods

### Sampling procedures

For bacterial data analysis, samples were collected from apple cultivar ‘Gala’ at the Michigan State University Plant Pathology farm. Harvesting was done on 9 September 2021. Six trees were selected at random, and 40 fruits per tree were sampled from around the circumference of the tree from each of six replicate trees. The fruits from each tree were maintained separately in clean plastic bins stored at room temperature in the laboratory. At each sampling point, five fruits were randomly selected from each replicate and pooled to make one biological replicate for a total of six biological replicates per time point. From each apple, two tissue types (skin and pulp) were sampled. Fruit pulp was excised using a sterile cork-borer and a sterile blade to peel a thin layer of the skin. Immediately, each sample tissue was homogenized separately in liquid nitrogen. The homogenized samples were stored at −80°C for subsequent DNA and RNA extractions. Samples were collected on day 0 (day of harvest), day 14, day 25, and day 49 after harvest.

For fungal data analysis, healthy fruits of apple cultivars ‘Gala’ and ‘Jonathan’ were collected at the Michigan State University Plant Pathology farm. Harvesting was done on different dates due to differences in ripening times between the two cultivars; ‘Gala’ was harvested on 9 September 2022, and ‘Jonathan’ on 7 October 2022. The same procedure was carried out for sample collection as mentioned above. However, the sampling time points were slightly different for the fungal data which were sampled from remaining apple fruits every two weeks until the 6^th^ week after harvest, after which, the final ‘rotten’ stage was identified when a lesion of at least 1 cm in diameter had appeared in at least 90% of the remaining fruits (day 96 and day 67 for Gala and Jonathan, respectively). At this last stage, samples were collected from intact tissue on a side of the fruit lacking any visible signs of necrosis.

### DNA extractions and sequencing of 16S rRNA and ITS genes

Microbial genomic DNA samples were extracted using the FastDNA SPIN Kit for Soil (MP Biomedicals, Solon, OH, United States) following the instructions of the manufacturer. Extracted DNA was used as the template for amplicon PCR reactions that amplified the bacterial 16S ribosomal region and fungal internal transcribed spacer (ITS) 1 region. The V4 region of the 16S rRNA was amplified using the universal 515F/806R primer set [48], and the ITS1 region was amplified using the ITS1F/ITS2 primer set [49, 50]. Peptide nucleic acid (PNA) clamps [51] were added to the 16S rRNA PCR mix (total of 20 μl) with a concentration of 2.5 μM mitochondrial PNA and 2.5 μM plastid PNA to block amplification of host plastid and mitochondrial 16S DNA. Raw sequence data preparation and data analysis were performed using Quantitative Insights into Microbial Ecology 2 (QIIME 2) [52]. Primer and adapter sequences were removed using cutadapt, paired reads were joined, samples were denoised with dada2, and chimeric sequences were removed using VSEARCH [53]. Operational taxonomic units (OTUs) were identified at 97% similarity using QIIME’s sklearn classifier and the SILVA database v132 for bacteria [54]. CONSTAX2 was used with the UNITE fungal general release dataset from 29 November 2022, to assign fungal taxa [55] with 80% confidence threshold and recommended settings [56]. Finally, sequences assigned to host mitochondria and chloroplasts were discarded.

**Table 1:** Gene Expression Targets for qPCR and their primer sequences

**Defense genes**	**Primers**	**Housekeeping genes**	**Primers**
***MdFLS2;*** Md05g1289000	*5’-TCCCTGCACGACAATGCTT* *5’-GTGGAATTGGACCCGTCAGT*	***Mdβ-ACTIN;*** Md01g1001600	*5’-CTATGTTCCCTCGTATTGCAGACC* *5’-GCCACAACCTTGTTTTTCATGC*
***MdBAK1;*** Md08g1221700	*5’-CGGGGAGCTACAGTTCCAAA* *5’-GCAGCCTTTCTGTTGGTGTC*		
***MdPR-4;*** Md04g1225400	*5’-GAAGGTGCCTCTTGGTG* *5’CGTCGGTGTCAATTTGG*		

### Data processing and analysis

The OTU table, taxonomy, metadata, and phylogenetic tree were imported into the R package Phyloseq v.1.24.2 [57]. Host mitochondria and chloroplast OTUs were removed. Alpha diversity was estimated with Shannon diversity index to determine the evenness based on the presence of rare OTUs (singletons and doubletons), respectively using Phyloseq v.1.24.2 [57]. Statistical significance was calculated by analysis of variance (ANOVA). Beta diversity was analyzed to compare the microbiome composition among groups, based on the Bray–Curtis dissimilarity distance matrix. The ordination was calculated by principal coordinates analysis (PCoA). To compare the microbiome composition between time points, statistical significance was calculated with permutational multivariate analysis of variance (PERMANOVA) using the vegan package v.2.6–4 [58]. To visualize the relative abundances of OTUs/ASVs, a bar plot was constructed using the ggplot2 package [59].

Differentially abundant taxa were identified using DESeq2 v.1.38.3 to extract the main effects of time point in each cultivar and tissue type [60, 61]. Taxa with similar trends in abundance over time were grouped using hierarchical and K means clustering. The elbow method was used to identify the optimal number of clusters. Abundance trends were visualized using pheatmap v.1.0.12 [62] and ggplot2 v.3.5.0. Indicator species analysis was performed using indicspecies v.1.7.14 to identify possible unique taxa and compositional signatures at each timepoint [63].

### Network construction and analysis

#### Data preprocessing

Microbial co-occurrence networks were constructed for ITS and 16S data for each cultivar × tissue type using abundance data from all timepoints for a total of six networks. For each subset, abundance data was filtered to remove any mitochondrial or chloroplast contaminants, OTUs that were not present in at least one sample, and samples with no reads. Because a low number of OTUs were significant by DESeq2, we included all OTUs in our network analysis.

#### Network inference

Processed abundance data and the corresponding metadata were used as input for network construction using FlashWeave (v0.19.2) [64] in Julia (v1.9.2) [65] with default parameters, selecting the sensitive mode to improve detection of weaker interactions and setting heterogeneous to false. Networks were saved as .gml files and imported to Cytoscape (v3.10.2) [66] for visualization and cluster analysis.

#### Network clustering

Networks were visualized using the yFiles Organic layout [67] and a network summary was generated using the Cytoscape tool ‘Analyze Network’ [68]. Network clusters were generated with clusterMaker2 [69, 70] using the MCL algorithm with inflation set to 2.5. New network visualizations were generated from the resulting clusters. Node and edge tables were exported for further analysis in R (v4.2.2).

#### Functional annotation

For bacterial communities, additional functional annotations were added in Cytoscape using microbetag [71] through the MGG app. Microbetag provides literature-oriented annotations using FAPROTAX, genomic-oriented annotations with phenDB, pathway complementarity between nodes using the KEGG database, and complementarity and competition seed scores between metabolic pathways using PhyloMInt. Although many of our OTUs were not annotated using this method, and therefore, we did not incorporate these annotations in downstream analysis, we provide the annotations in Table S3.

#### Network analysis

Nodes linked to OTUs and metadata were separated and taxonomic information, DESeq2 results, and Indicator Species analysis were merged with the network properties. Nodes were considered network hubs if their degree was greater than 2.5 (overall average degree for each network was close to 2), network connectors if their betweenness centrality was greater than 0.65, and keystone taxa if their degree was greater than 2.5, and their betweenness centrality was greater than 0.65. Nodes were additionally annotated if they were identified as an indicator species at any timepoint or significant by DESeq2 at any timepoint. To visualize relative abundance of taxa annotated as hub, connector, or keystone across timepoints, technical replicates from DESeq2 normalized abundance data were averaged and percent relative abundance was calculated for each timepoint.

Edges were divided into positive and negative associations and plotted using circlize (v0.4.16) [72] to visualize connections between phyla. All other visualizations were produced using ggplot2 (v3.5.1) [59].

#### Statistical analysis

Overall network properties were summarized using Cytoscape and compared qualitatively. Degree and betweenness centrality were compared across phyla for each network using ANOVA followed by the Tukey HSD *posthoc* test as appropriate in the R stats package [73]. Connecting letter reports were generated using multcompView (v0.1-10) [74]. The Fisher Exact Test followed by Benjamini–Hochberg correction from the R stats package [73] was used to determine if any phyla were enriched for hub, connector, or keystone nodes.

Additional packages for data processing, analysis, and visualization include dplyr (v.1.1.4) [75], tidyr (v1.3.1) [76], openxlsx (v4.2.5.2) [77], tidyverse (v2.0.0) [78], stringr (v1.5.1) [79], ggrepel (v0.9.5) [80], and RColorBrewer (v1.1–3) [81].

### RT-qPCR analysis

Expression of host defense genes (Table 1) was monitored at three time points for Jonathan and four time points for Gala. This experiment was done on the samples collected from 2022. Samples from the sampling time point ‘Rotten’ was collected from an intact tissue with no lesion. RNA extraction (RNeasy plant mini kit- QIAGEN), and reverse-transcription (SYBR Green Real-Time PCR master mix), were performed according to the manufacturer’s instructions. Real-time qPCR was performed with 35 cycles using a set of primers for each of the defense genes. Real-time qPCR reaction for *Mdβ-ACTIN* and *MdPR-4* were prepared as described in [82, 83], respectively. Additional information such as the accessions and primer sequences are indicated in Table 1. The expression of defense marker genes was normalized to the expression of the *Mdβ-ACTIN* gene. Significant differences (*P* < 0.05) were calculated using one-way ANOVA.

### flg22 treatment and *P. expansum* pathogenicity assay

Healthy fruits of apple cultivars ‘Pink Lady’ were collected at the Michigan State University Plant Pathology farm. Harvesting was done on 22 October 2022. Apples have a limited time period for harvest, and once we miss that window of time, the fruit will become overripe and more susceptible to pathogens during storage. Since we wanted to conduct this experiment on freshly picked apples when they have a stable and robust microbiome and immune response, respectively, Pink Lady was used as they were at the correct stage for harvest, and we had missed the harvest stage for Gala and Jonathan.

On the day of harvest, each freshly harvested ‘Pink Lady’ apple was wounded with a sterile needle with 15 injuries randomly around the fruit (each 1 × 3 mm). The wounded apples were dipped for 1 min in 2.5 μmol flg22 solution containing the organosilicone surfactant Silwet L-77 (Helena Agri-Enterprises, Collierville, TN), at a concentration of 0.025%. Control apples were wounded, but only dipped in 0.025% Silwet L-77. Samples (both skin and pulp) were collected for RNA extraction at zero-, one-, two-, four-, six-, twelve-, and twenty-hour post treatment. At each sampling point, three fruits were randomly selected and pooled to make one biological replicate for a total of three biological replicates. For RT-qPCR, transcript levels were normalized to that of the house-keeping gene *Mdβ-ACTIN*.

A fungal pathogenicity assay was conducted using six apples (biological replicates) per treatment. Fifteen μl of a *P. expansum* spore suspension (1 × 10^4^ conidia/mL) was inoculated on two opposite sides of each wound. Conidia suspensions were made in 0.05% (w/v) Tween-20. Control samples were inoculated with 15-μl sterile water containing 0.05% (w/v) Tween-20. After air-drying, the fruits were placed in a clean bin without the fruits touching each other. The bins were then covered with plastic film to maintain a high relative humidity of 80–90% and incubated at 20 ± 1°C. Lesion diameters (cm) were regularly monitored and measured when symptoms emerged.

## Supplementary Material

Web_Material_uhaf063

## Data Availability

Raw data used in this study are available in the NCBI Sequence Read Archive (SRA) under the BioProject IDs: PRJNA1112867 and PRJNA1112895.
