# Supplementary figures and images for: Temporal fruit microbiome and immunity dynamics in postharvest apple (Malus x domestica)
Source: Hortic Res. 2025 Feb 25;12(6):uhaf063. doi: 10.1093/hr/uhaf063 (PMC12023859; doi:10.1093/hr/uhaf063)

(a)

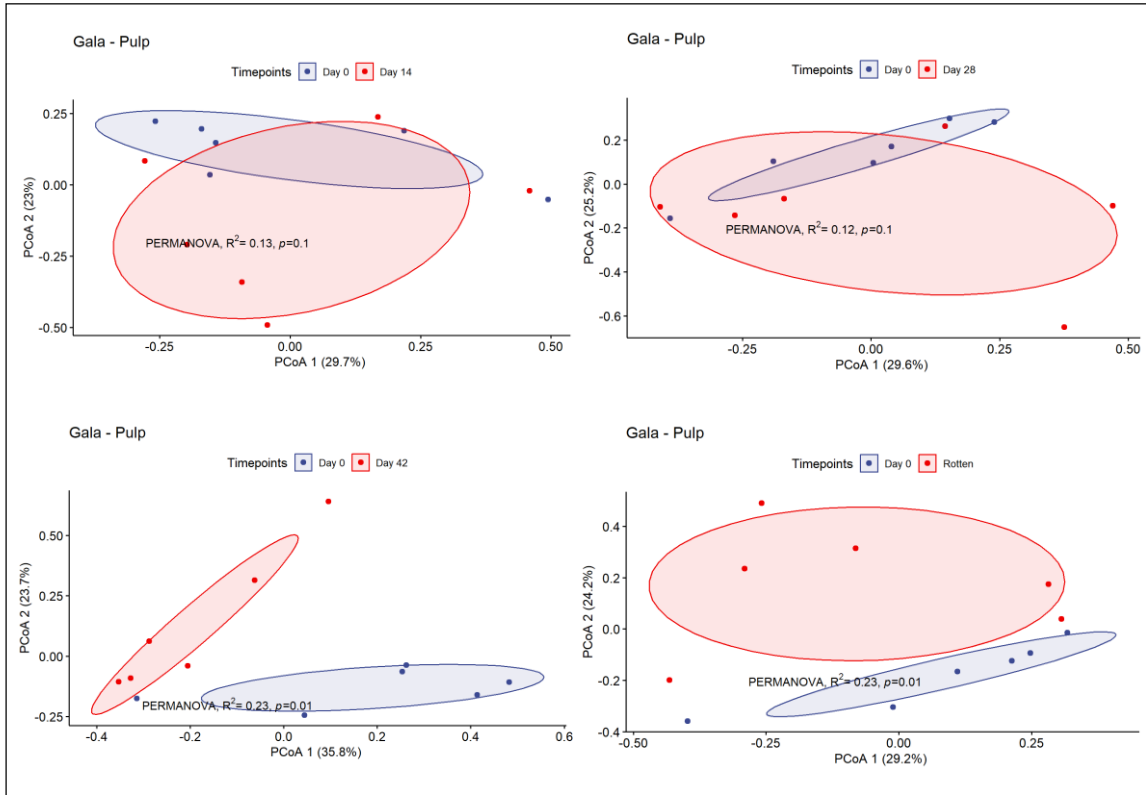

(b)

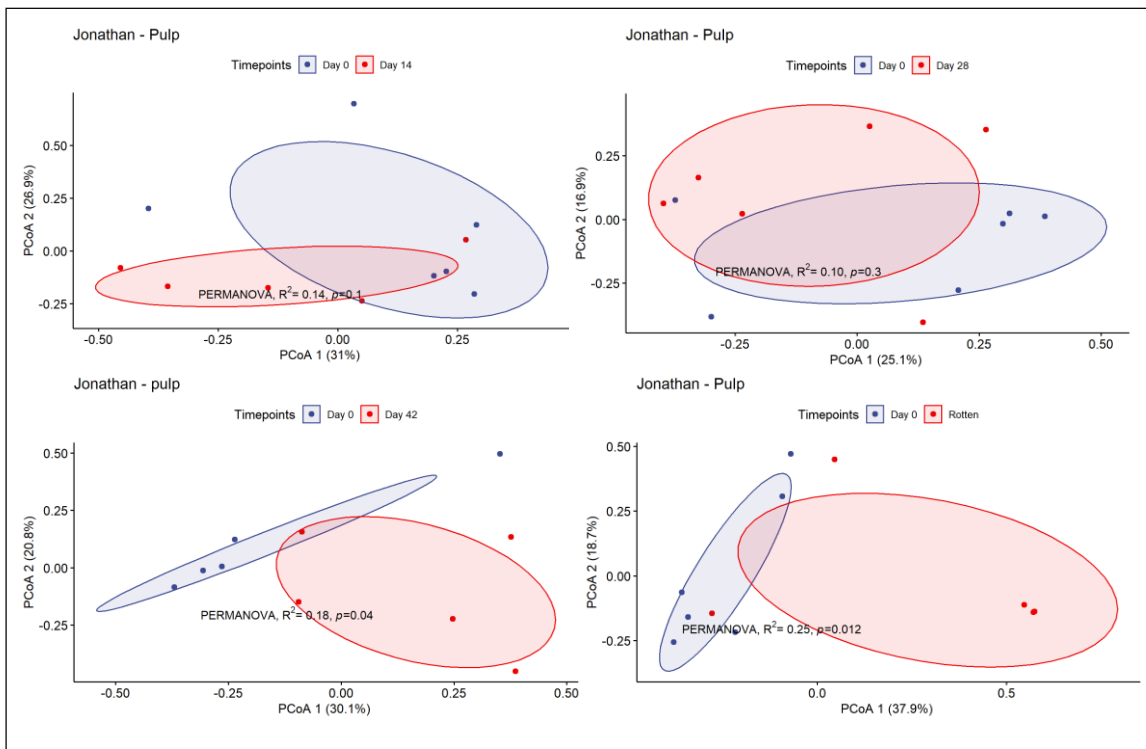

(a)

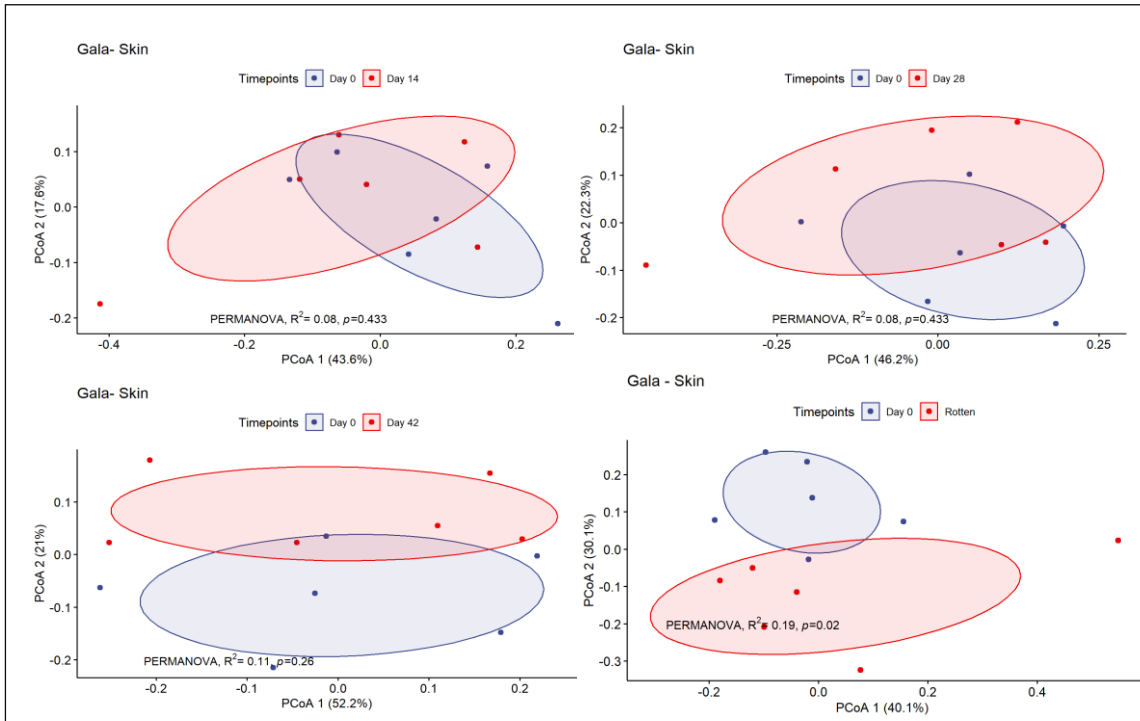

(b)

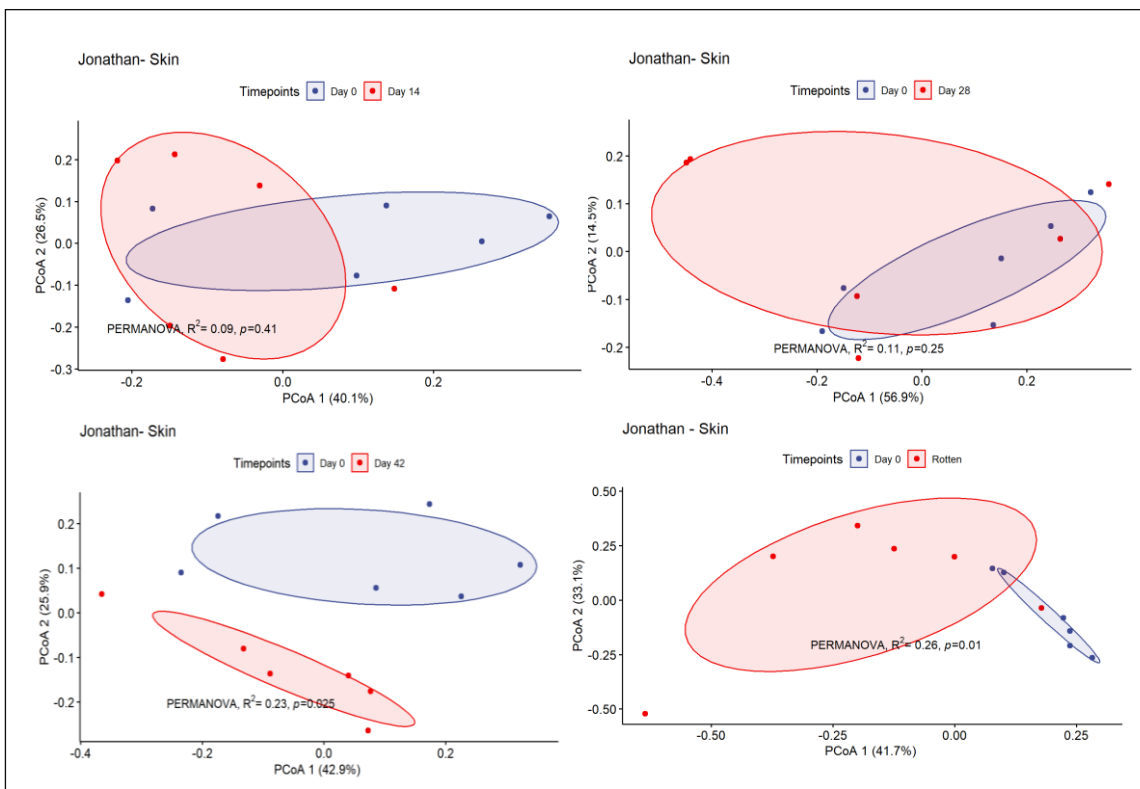

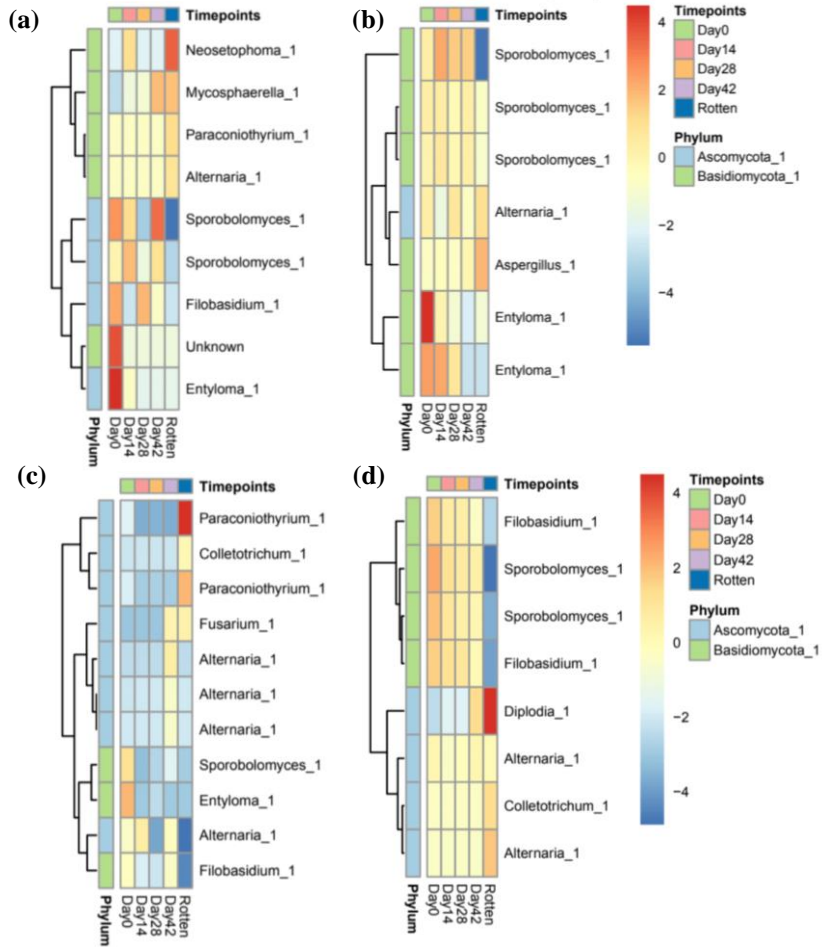

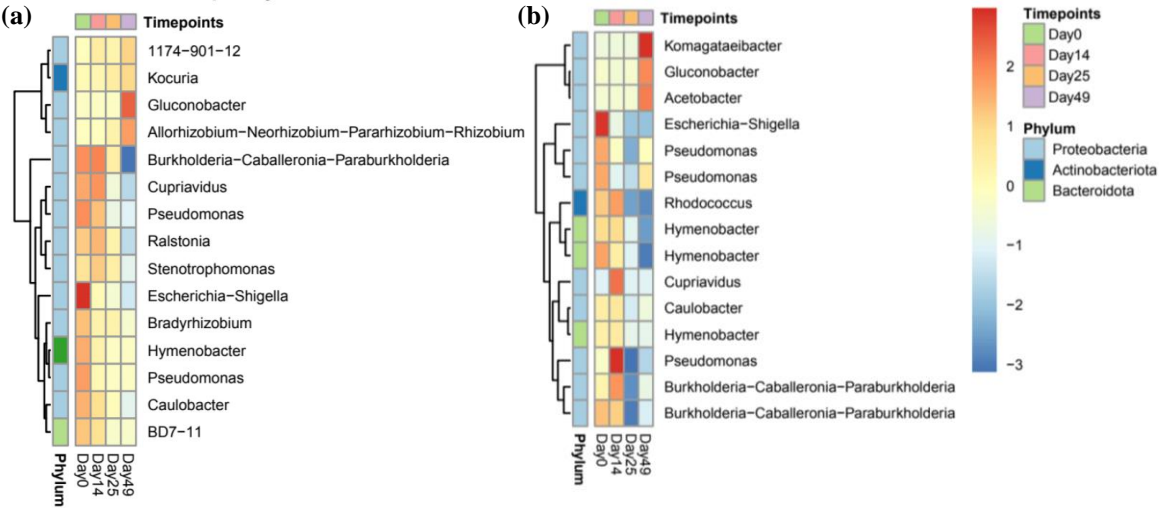

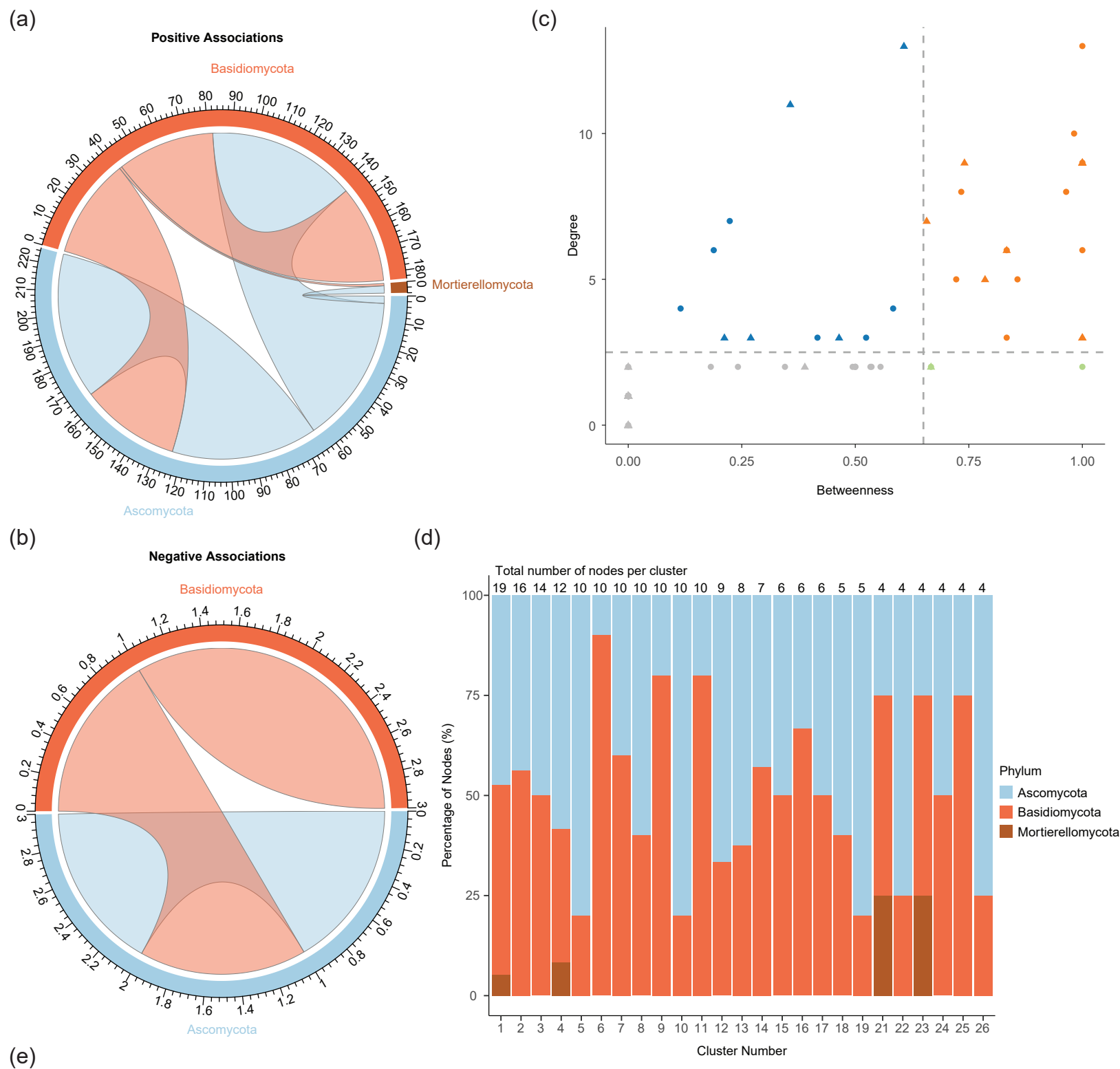

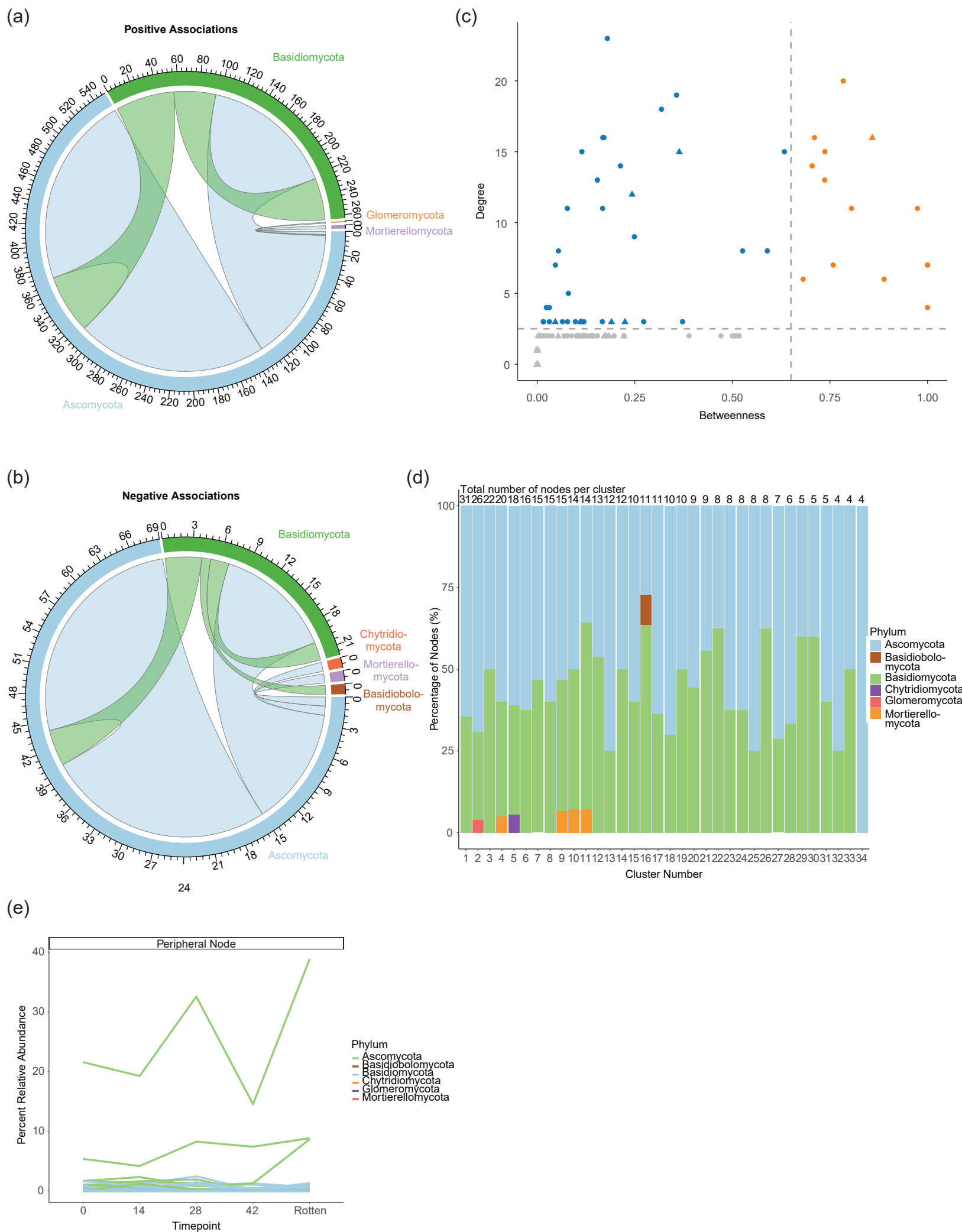

(a)

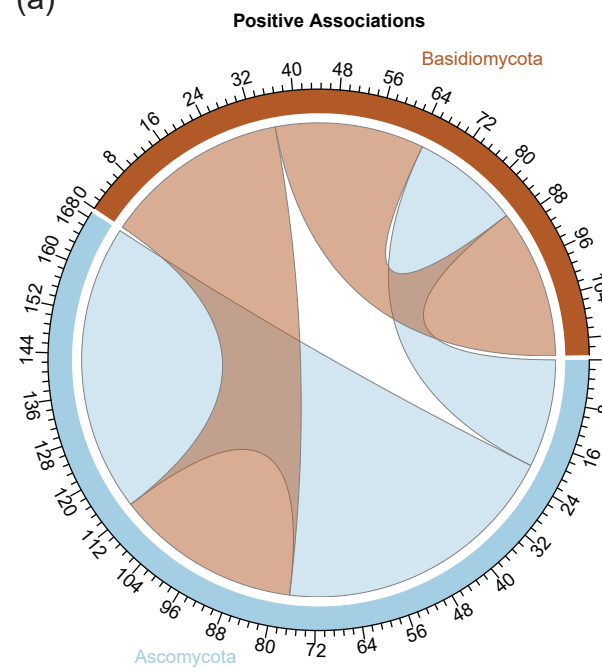

(c)

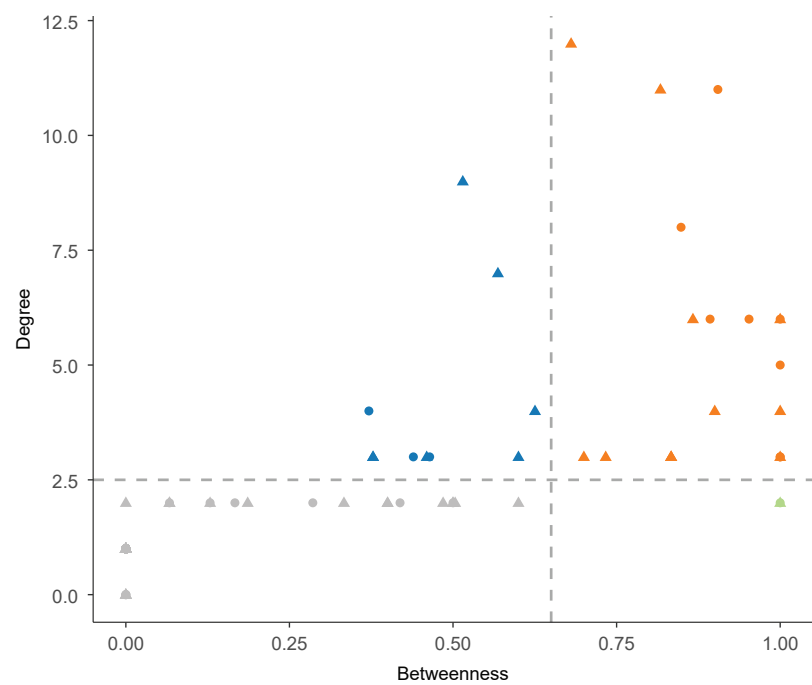

(b)

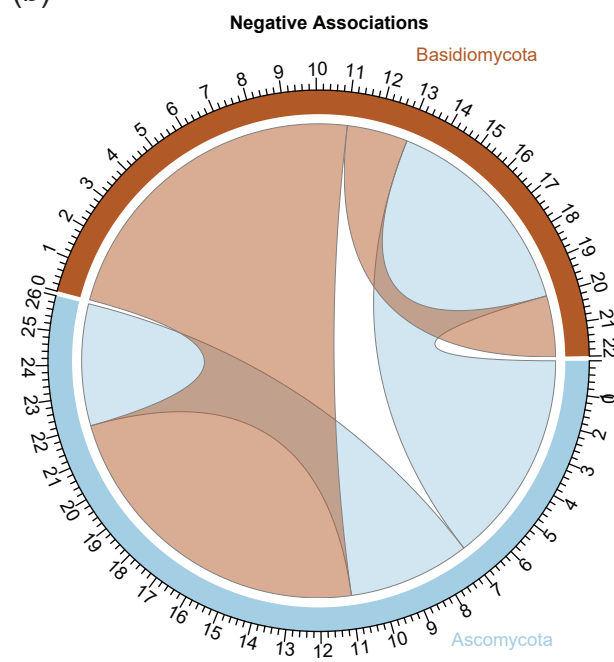

(d)

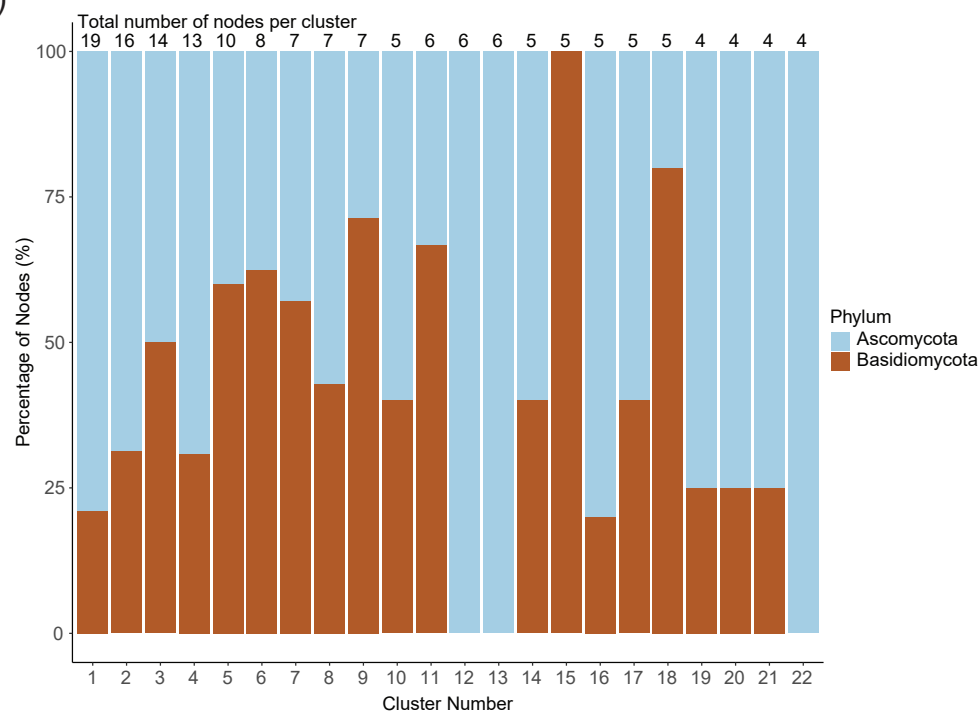

(e)

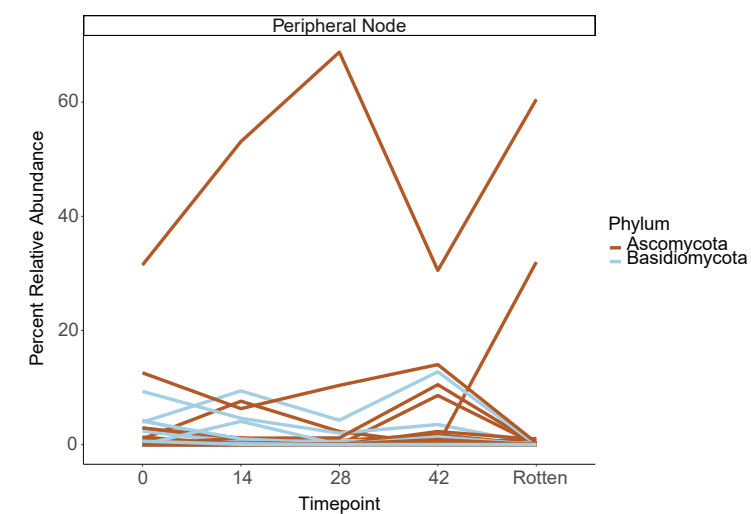

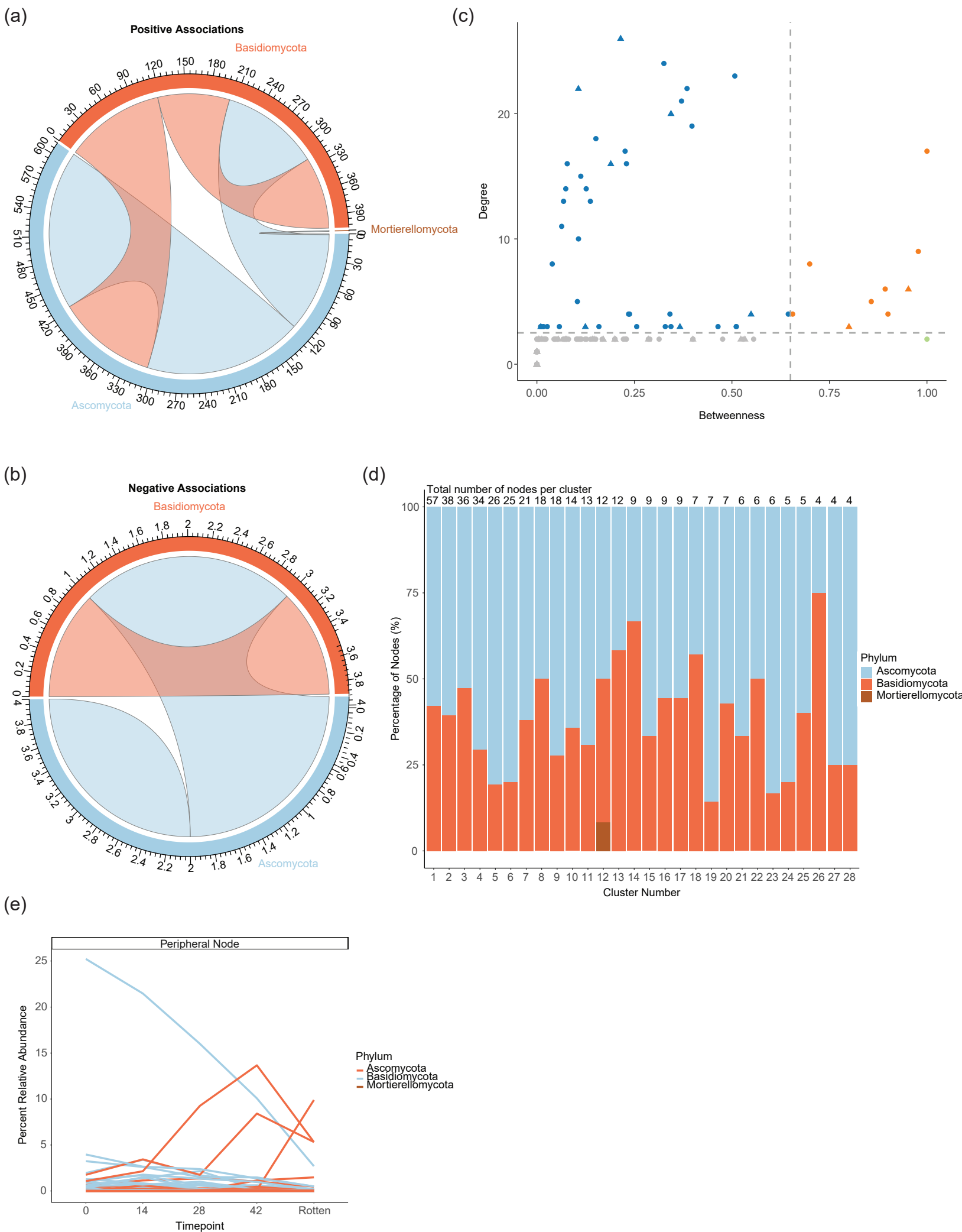

(a)

## Positive Associations

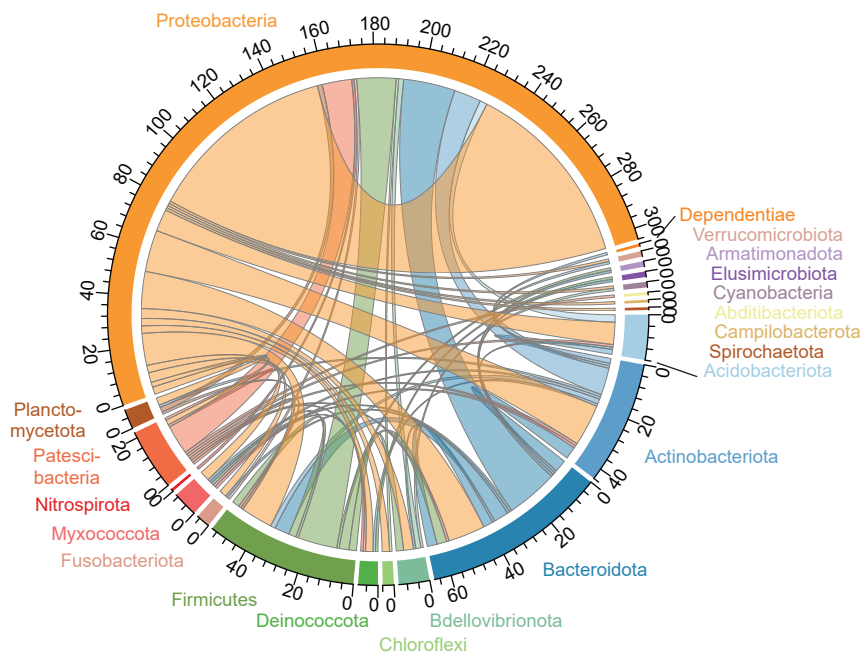

(b)

## Negative Associations

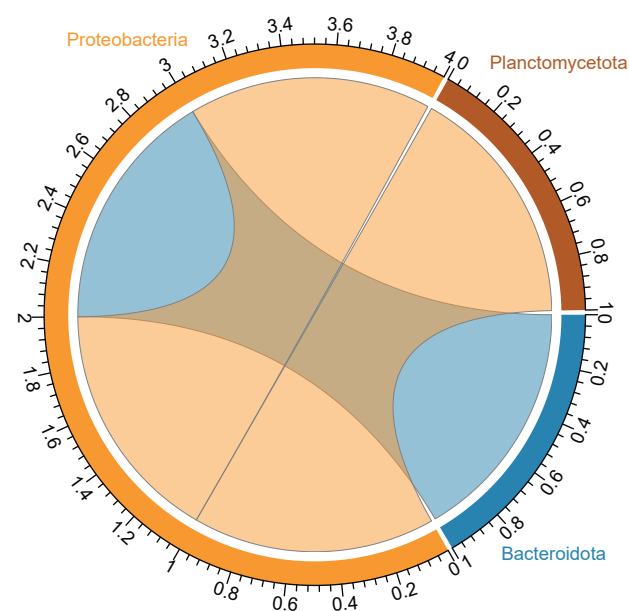

(c)

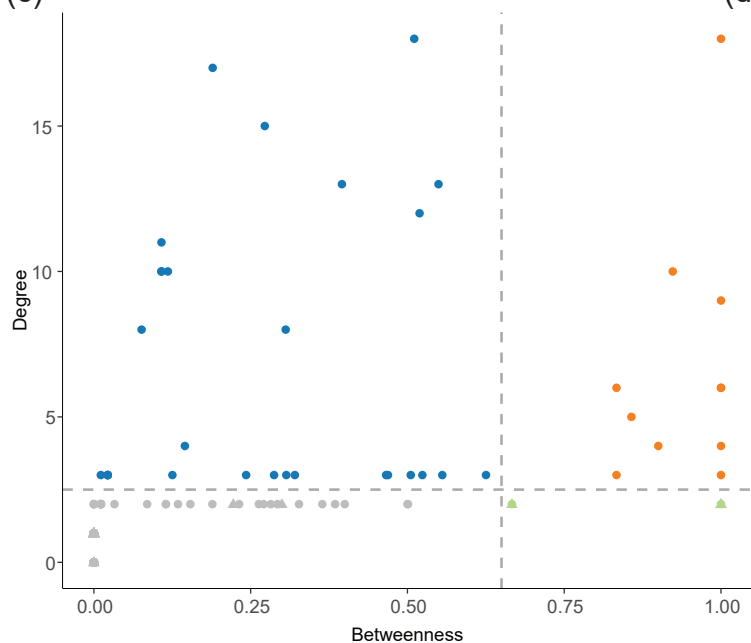

(d)

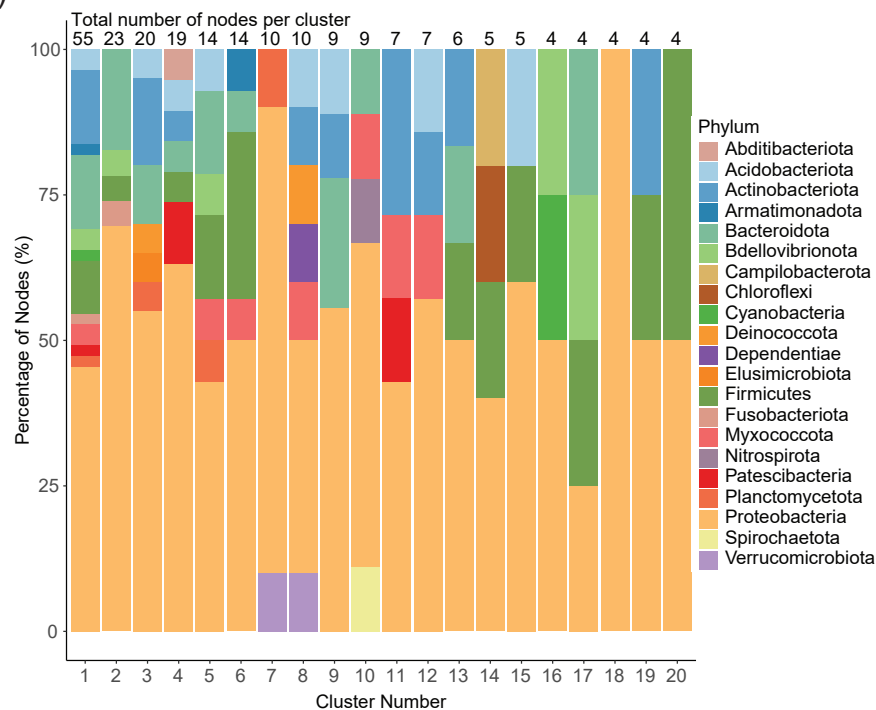

(e)

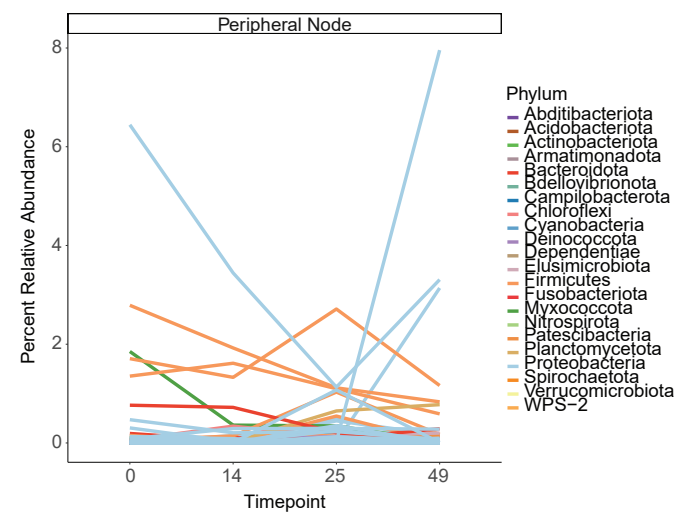

(a)

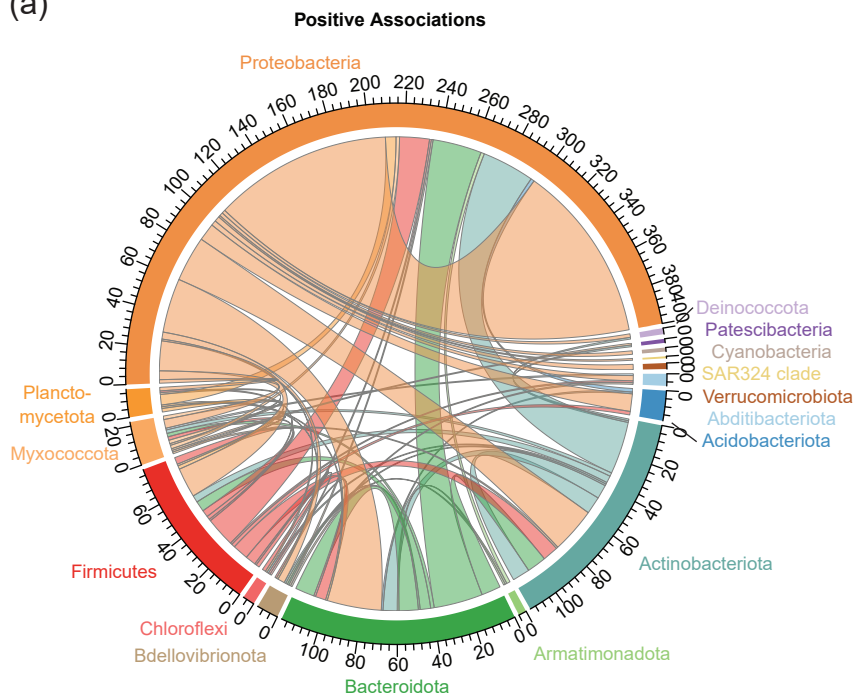

(b)

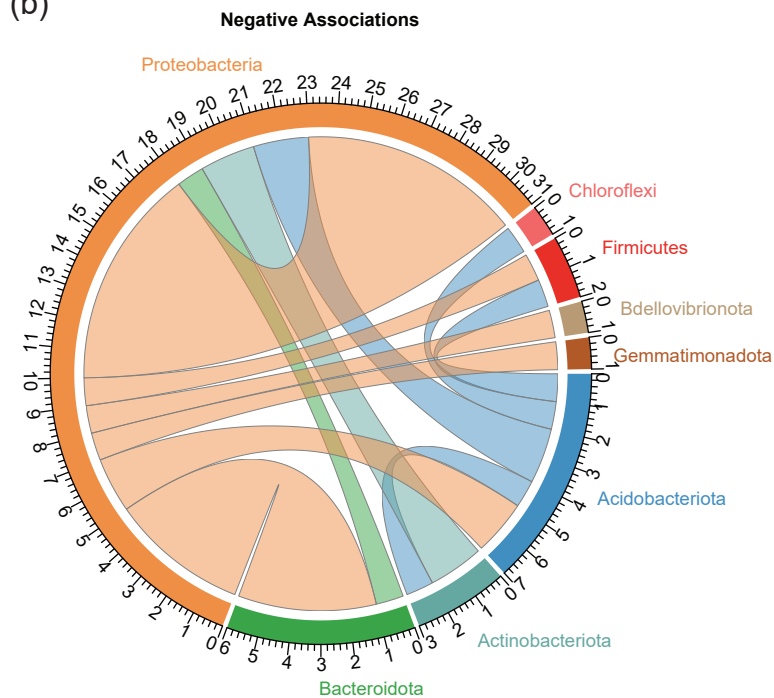

(c)

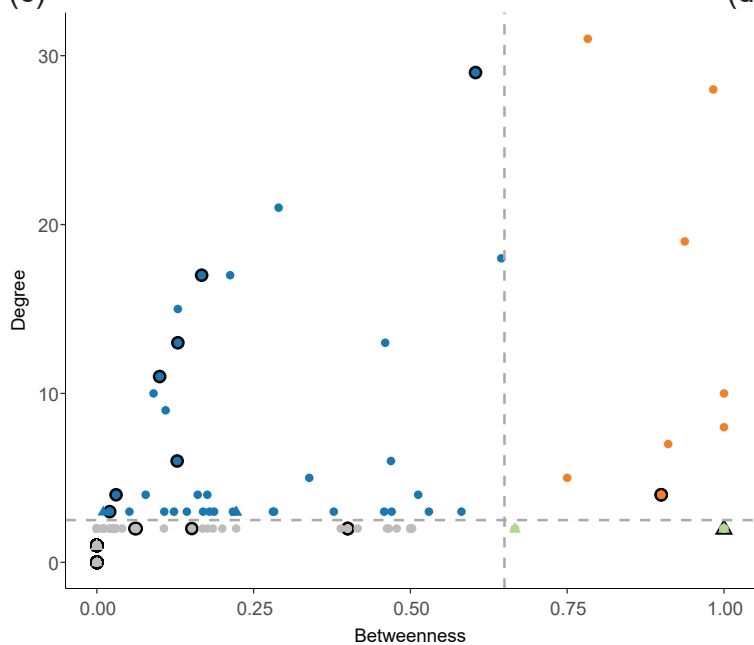

(d)

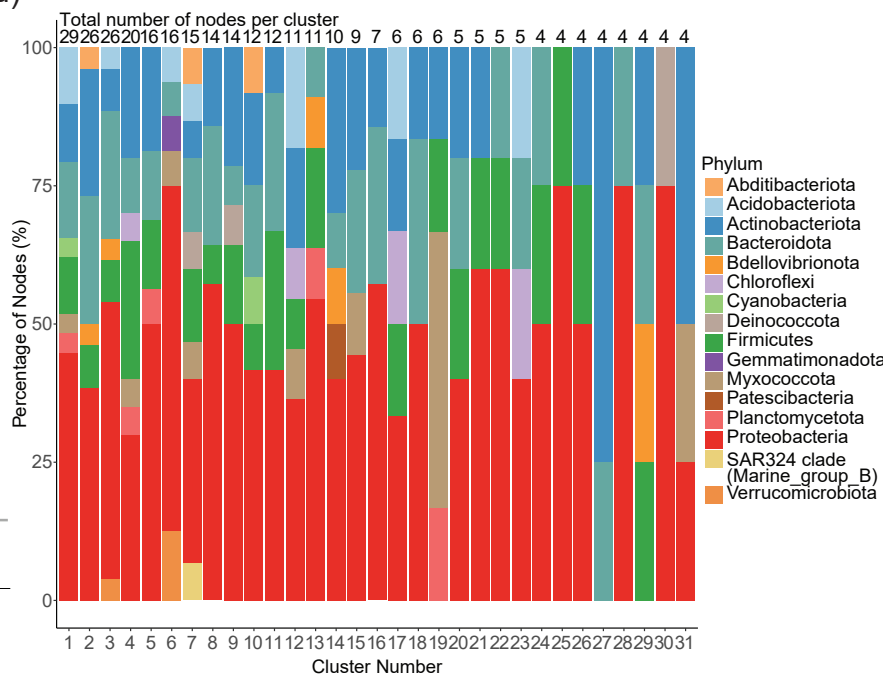

(e)

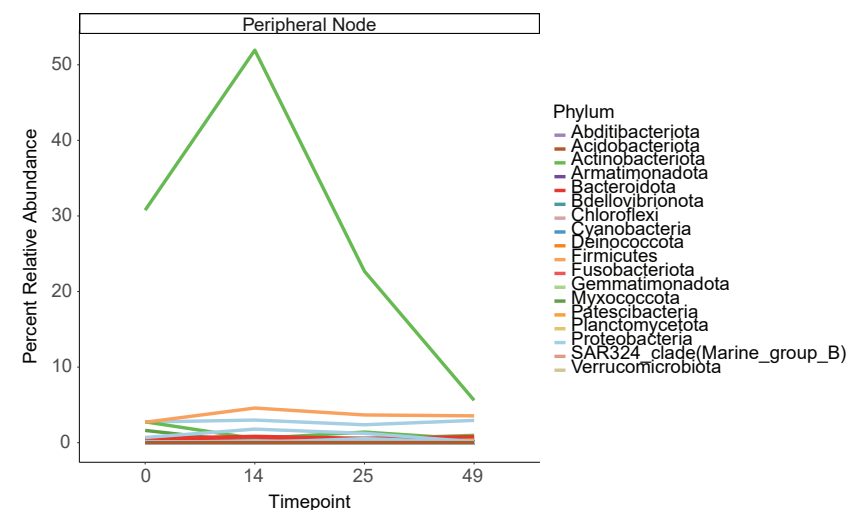

Supplement: Web_Material_uhaf063 [file web_material_uhaf063.zip › Kithan et al Suppl figures 1-10.pdf]
